# Real-time sensing-integrated organoid-on-a-chip platforms: Technological progress and emerging biomedical applications

**DOI:** 10.1016/j.bioactmat.2026.06.041

**Published:** 2026-07-04

**Authors:** Chenwei Sun, Guohua Wu, Di Wu, Qijun Du, Qingrui Lu, Wenqi Hu, Jiashu Wang, Ao Xie, Zipeng Yao, Mengjiao Xia, Haijie Hu, Shuqi Wang

**Affiliations:** a College of Biomedical Engineering, Sichuan University, Chengdu, 610065, China; b National Engineering Research Center for Biomaterials, Sichuan University, Chengdu, 610065, China; c Luoyang Key Laboratory of Clinical Multiomics and Translational Medicine, Henan Key Laboratory of Rare Diseases, Endocrinology and Metabolism Center, The First Affiliated Hospital, College of Clinical Medicine of Henan University of Science and Technology, Luoyang, 471003, China; d Department of Respiratory and Critical Care Medicine, West China Hospital, Sichuan University, Chengdu, 610041, China; e State Key Laboratory of Respiratory Health and Multimorbidity, West China Hospital, Sichuan University, Chengdu, 610041, China; f Tianfu Jincheng Laboratory, City of Future Medicine, Chengdu, 641400, China; g Division of Biliary Surgery, Department of General Surgery, West China Hospital, Sichuan University, Chengdu, 610041, China; h Clinical Research Center for Respiratory Disease, West China Hospital, Sichuan University, Chengdu, 610041, China

**Keywords:** Organoid-on-a-chip, Real-time sensing, Microenvironmental engineering, Clinical precision medicine, Closed-loop feedback

## Abstract

As micro-scale 3D tissues self-organized from stem cells, organoids can highly recapitulate the cellular composition and complex spatial architecture of human organs, establishing themselves as pivotal physiological models in biomedical research. Although organoids offer significant advantages in mimicking human physiological structures, traditional monitoring methodologies predominantly rely on destructive endpoint assays, which fail to capture the transient fluctuations inherent in biological processes. To overcome this limitation, we propose the sensing-integrated organoid-on-a-chip, a frontier interdisciplinary platform. This review systematically outlines the comprehensive construction of this platform, focusing on the synergistic integration of microenvironmental engineering and real-time sensing technologies. The article provides an in-depth analysis of real-time monitoring facilitated by high-performance electrical, optical, and mechanical sensors to quantify organoid developmental maturation, metabolic fluctuations, and pathological evolution. We emphasize the application potential of this platform across developmental biology, disease modeling, drug screening, and neuroscience exploration. Furthermore, we discuss the integration of closed-loop feedback regulation systems and artificial intelligence-assisted analysis, while outlining the trajectory of this platform toward clinical precision medicine and industrial standardization. We firmly believe that sensing-integrated organoid-on-a-chip platforms will accelerate the advancement of personalized diagnosis and therapeutics, thereby ushering in a new era of dynamic biomedical research and intelligent healthcare.

## Highlights

• Systematically summarizes advanced strategies for engineering organoid-on-a-chip platforms by integrating microfluidic regulation, bioactive matrix design, and multimodal physicochemical stimulation.
• Provides a comparative analysis of electrical, optical, and mechanical monitoring technologies, emphasizing their efficacy in capturing transient metabolic fluctuations and bioelectric signatures during organoid development and pathological progression.
• Elucidates how real-time sensing transforms organoids from static models into dynamic predictive tools for applications in developmental biology, disease modeling, precision oncology drug screening, and neural activity monitoring.
• Offers perspectives on the integration of closed-loop feedback systems, high-fidelity biological assembloids, and artificial intelligence, alongside the potential for industrial standardization to facilitate clinical translation.

## Graphical abstract

Schematic representation of the sensing-integrated organoid-on-a-chip platform spanning from advanced multi-modal modulation to real-time analysis and biomedical applications. The platform achieves high-fidelity microenvironmental engineering through precise biophysical and biochemical stimulations within microfluidic cellular scaffolds. Concurrently, integrated bio-sensors enable the continuous, non-destructive, and real-time tracking of transient organoid physiological trajectories via electrophysiological, electrochemical, optical, and mechanical monitoring modalities. This paradigm effectively drives the advancement of downstream frontiers, including developmental biology, pathological mechanism exploration, drug screening, and consciousness activity monitoring.

**Figure:**
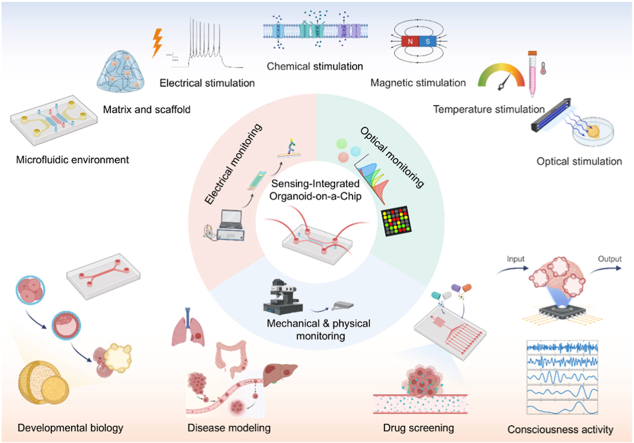

## 1 Introduction

Understanding how organs form, mature, and dynamically function across the human lifespan has long been a central pursuit in biomedical research, with decades of studies in model organisms such as mice, zebrafish, and Drosophila, and in vitro embryonic stem cell systems [1,2]. Traditional 2D cell cultures [3], organ-on-a-chip [4] and animal models [5] have been instrumental in uncovering fundamental biomedical mechanisms. However, 2D systems lack the structural complexity and integrated signaling networks required for physiologically relevant responses [6], and animal models often exhibit interspecies discrepancies in gene regulation, metabolism, and tissue organization [7], limiting translational relevance. In contrast, 3D organoids, self-organized structures derived from stem cells or primary tissues [8,9], can recapitulate multicellular compositions, microanatomical features, and organ-specific functions in vitro [[10], [11], [12]], offering enhanced potential for drug screening, disease modeling, regenerative medicine, and personalized therapy. The biological fidelity and functional complexity of these models depend fundamentally on the initial cellular source [13,14]. Primary tissue-derived cells provide the most accurate reflection of a patient's immediate physiological state and typically contain specialized resident stem cell populations; however, their utility is often restricted by limited expansion capacity and donor scarcity. In contrast, renewable systems, most notably induced pluripotent stem cells, provide an inexhaustible cellular supply that is highly amenable to precise genomic editing. Within these systems, differentiation potential is organized in a hierarchical manner. This hierarchy extends from totipotent and pluripotent stem cells such as embryonic and induced pluripotent stem cells, which possess the capacity to differentiate into all germ layers, to multipotent adult stem cells and finally to unipotent stem cells restricted to specific lineages. The distinction between 3D organoids derived from primary sources and those from stem cells lies primarily in their developmental maturity and proliferative potential. Consequently, the strategic selection of a specific cell type remains the decisive factor in constructing biological models.

Beyond developmental biology, gaining real-time insight into how tissues maintain homeostasis, adapt to physiological stimuli, transition into pathological states, and respond to therapeutic interventions is essential for advancing disease modeling, drug discovery, and regenerative medicine [15,16]. However, most conventional in vitro and in vivo methodologies provide only static, end-point, or low-frequency readouts, including histology, immunostaining, and secretome analysis [[17], [18], [19]], making it difficult to capture the rapid biochemical, electrophysiological, and metabolic fluctuations that underpin these dynamic processes [20]. These challenges are further compounded by the inherently nonlinear, stage-dependent, and often stochastic nature of biological processes, in which functional outcomes frequently emerge from temporal patterns, transient fluctuations, and early-phase responses rather than terminal values. The lack of continuous monitoring not only prevents timely adjustment of culture conditions but also obscures sample-specific developmental or metabolic trajectories, undermining reproducibility and comparability across experiments. These limitations underscore the need for platforms that integrate physiologically relevant 3D human tissue models with high-resolution, real-time sensing, enabling dynamic characterization, interpretation, and regulation of organ-level behavior. These considerations have spurred the development of 3D organoid-on-a-chip platforms that combine physiologically relevant tissue models with real-time monitoring capabilities, enabling dynamic control and functional readouts while the technology continues to mature.

In recent years, with the advancement of miniaturized electrochemical sensors, integrable microelectrode arrays, multimodal optical imaging, spectroscopic technologies, and mechanically responsive readout units, organoid-on-a-chip platforms have acquired the capability for real-time, non-destructive, and multidimensional monitoring. This provides unprecedented, high-temporal-resolution tools for elucidating organ developmental dynamics, pathological progression, and drug responses. However, existing reviews predominantly classify and summarize individual monitoring techniques or summarize organ-specific design strategies [[21], [22], [23], [24]], leaving a void in the systematic discussion of their cross-domain applications. Recently, Kong et al. [25] systematically reviewed organ-on-a-chip technologies from macro-engineering perspectives, including microfluidic fabrication, pumping and pumpless systems, commercial validation, and space biology. While that work offers visionary guidance by providing an engineering overview for the field, its discussion on sensing integration remains primarily focused on high-level application showcases. Nevertheless, the underlying materials-science logic of real-time monitoring technologies, such as how electrode material selection affects signal stability, how optical window materials limit imaging depth, and how flexible or stretchable materials determine the sensitivity of mechanical sensing, has not been sufficiently reviewed. Crucially, existing literature fails to address the reciprocal relationship between the physical limitations of biomaterials and the diagnostic fidelity of biological signals. There remains a significant knowledge void regarding how the spatiotemporal mass-transport limits and structural compliance boundaries of culture matrices directly govern the signal-to-noise ratio, baseline stability, and spatial parameters of integrated transducers. Furthermore, a systematic synthesis is still lacking regarding the materials-level architecture required to achieve non-interfering synchronization between active microenvironmental modulation, such as biophysical or biochemical stimulation, and in situ real-time sensing, leaving the operational principles of adaptive, closed-loop platforms largely fragmented.

**Fig. 1:**
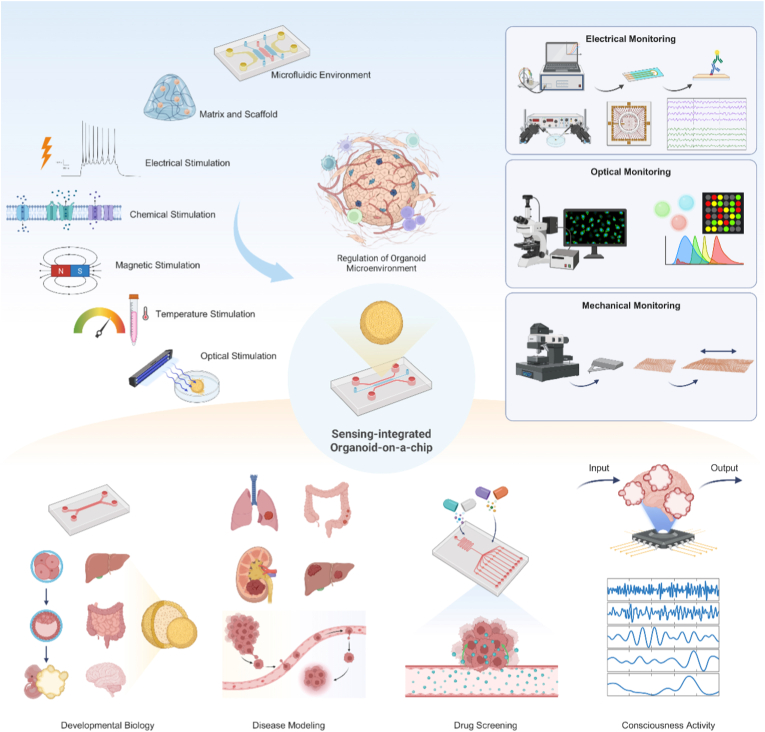
Schematic overview of sensing-integrated organoid-on-a-chip platforms from microenvironment engineering and multi-modal monitoring towards biomedical applications. The platform regulates the organoid microenvironment through diverse physical and chemical stimuli (left), including electrical, chemical, magnetic, thermal, and optical modulations within microfluidic scaffolds. Integrated sensor arrays enable real-time tracking of organoid behaviors via electrophysiological, optical, and mechanical monitoring systems (right). The technological advances are applied in downstream biomedical research (bottom), including developmental biology, disease modeling, drug screening, and the decoding of neural network dynamics during cognitive computing and consciousness activities.

To address these gaps, this review systematically summarizes recent advancements in organoid-on-a-chip construction and microenvironmental modulation. The core innovation and unique contribution of this work, distinguishing it from existing published reviews, lies in its systematic combination of material science and sensing biology, offering a crucial conceptual perspective that correlates culture matrix properties with real-time sensing performance. We further provide a comprehensive, dual-perspective evaluation, spanning materials science and organoid biology, of the integrability, applicability, and limitations of electrical, optical, and mechanical monitoring technologies across diverse organoid types, with a particular focus on the coupled design between the matrix microenvironment and sensing performance. Moreover, we highlight the multidimensional utility of organoid-on-a-chip systems in metabolic analysis, disease modeling, drug screening, and the exploration of neural activity [26] and consciousness-related signatures [27], while systematically addressing the critical interfacial issues and emerging directions in closed-loop regulation [28] and intelligent adaptive monitoring [29]. By establishing a cohesive framework that spans microenvironment engineering, sensor-material integration, and real-time functional readouts, this review offers a foundational framework for advancing, standardizing, and ultimately translating organoid-on-a-chip technologies toward clinical and industrial applications (Fig. 1).

## 2 Engineering advances in organoid-on-a-chip platforms

The functional maturation and structural organization of organoids typically follow two distinct engineering strategies [30]. The first approach is cell self-assembly. This method relies on the intrinsic genetic programs of cells to drive spontaneous organization into complex architectures [31]. Self-assembly excels at recapitulating natural tissue development and biological fidelity. However, this process often results in significant variability in organoid size and morphology. The second approach is template or matrix driven engineering. This strategy utilizes external cues such as biochemical, mechanical, and spatial signals required for tissue specific development [32,33]. These methods provide superior control over the physical dimensions and spatial orientation of the tissue model. These techniques are particularly advantageous for sensing integrated platforms. They enable the precise modulation of organoid microenvironments [34], microfluidics, engineered culture matrices, and physical stimuli. Such control ensures proper organoid development [9,35] while facilitating stable signal acquisition.

### 2.1 Microfluidic regulation of biochemical and biophysical microenvironment

As 3D structures formed through the self-organization of multiple cell types, organoids rely heavily on a microenvironment that can recapitulate in vivo physiological conditions to support long-term culture, functional maintenance, and structural integrity. Such conditions include stable nutrient supply, efficient metabolic waste removal, appropriate mechanical cues, and tissue-scale mass transport. However, conventional static culture systems are unable to satisfy these requirements simultaneously, often resulting in uneven nutrient gradients, hypoxia, structural collapse, and functional decline. The introduction of microfluidic technology provides a critical solution to these limitations. By precisely regulating hydrodynamic parameters such as flow rate, shear stress, concentration gradients, and dissolved oxygen levels, microfluidic platforms can create a highly controlled and continuously refreshed dynamic culture microenvironment that preserves organoid architecture while promoting cellular differentiation, functional maturation, and even vascularization. Thus, microfluidic regulation has become a central strategy in organoid-on-a-chip technologies for reconstructing physiologically relevant microenvironments and enabling more accurate modeling of organ development and disease processes.

For example, the role of microfluidics in supporting organoid spheroid growth, Huang et al. [36] developed a multi-system interactive microfluidic intestinal organoid chip capable of precisely regulating flow rate and oxygen concentration. This platform reproduced the rapid oxygen dynamics characteristic of intestinal ischemia-reperfusion injury and revealed metabolic, inflammatory, and apoptotic responses of organoids under hypoxia and reoxygenation, while simultaneously enhancing oxygen exchange efficiency. This highlights how fluid dynamics and oxygen supply can modulate the cell behavior in organoids and influence their maturation. Microfluidic regulation is also critical for vascular network formation. Quintard et al. [37] constructed a microfluidic platform that employs hydrodynamic trapping to position organoids with high spatial precision. Continuous perfusion within the device mimics in vivo nutrient and gas exchange, supporting endothelial self-assembly into vascular networks and markedly improving organoid growth, maturation, and physiological function. Further supporting the role of fluid flow in organoid vascularization, Kimberly A. Homan et al. [38] showed that applying fluid shear stress to kidney organoids during microfluidic culture promotes endothelial progenitor expansion and vascular plexus formation, enabling podocytes and tubule-like structures to acquire mature polarity and adult-like gene expression patterns reminiscent of embryonic kidney development. This highlights the critical role of fluid dynamics in mimicking physiological forces that drive vascular development and enhance organoid functionality.

To highlight the influence of fluid dynamics on organoid function, Cho et al. [39] engineered a platform integrating brain extracellular matrix with a microfluidic perfusion system. By applying cyclic medium flow, the device simulates physiological shear stress and dynamic nutrient transport in brain tissue. The resulting mechanical and biochemical cues significantly enhanced neurogenesis within brain organoids and promoted the organization of cortical layer structures, yielding more mature and orderly cortical hierarchies. This dynamic flow enhances oxygen delivery and metabolite exchange by creating concentration gradients across the organoid, mimicking the in vivo process of nutrient and waste transport. The continuous movement of the medium helps to maintain an optimal environment for cell metabolism, preventing the accumulation of metabolic waste and promoting efficient nutrient uptake. Cyclic medium flow also mimics the shear stress experienced by endothelial cells in vivo, which in turn influences cellular differentiation and vascularization, crucial for functional maturation of the organoid. Dynamic perfusion further improved oxygen and nutrient homogeneity, allowing for more uniform oxygen distribution and reducing regions of hypoxia. This not only reduces inter-organoid variability but also increases consistency in organoid size, cellular composition, and electrophysiological activity. The steady flow of nutrients and oxygen, combined with the removal of metabolic byproducts, ensures a more stable and reproducible experimental condition. To further refine these biophysical settings, the incorporation of numerical computational fluid dynamics (CFD) with fluid-structure interaction modeling has emerged as a milestone paradigm to establish highly precise hemodynamic boundaries within chips. For instance, Zhao et al. [40] introduced an aortic dissection chip coupled patient-specific CFD simulations with microfluidic execution to replicate precise wall shear stress profiles, successfully triggering smooth muscle cell pathological phenotypic transitions via the mechanosensitive PIEZO1 pathway. This computational-experimental synergy successfully breaks the bottleneck of clinical WSS non-quantifiability, offering a high-fidelity strategy for mechanism-driven vascular disease modeling and potential therapeutic target discovery.

The benefits of dynamic perfusion extend beyond normal tissue organoids to disease models, particularly in recapitulating the complex tumor microenvironment. To provide a more physiologically relevant foundation for tumor organoid modeling, Benjamin et al. [41] integrated patient-derived pancreatic tumor organoids with a microfluidic vascular model, successfully reconstructing an in vitro tumor microenvironment. This platform recapitulated the influence of vascular networks on tumor growth and invasion, offering a more biologically relevant system for mechanistic studies. Beyond supporting structural and functional maturation, microfluidics also enables spatial and temporal control of drug or environmental chemical exposure, thereby advancing pharmacokinetic and toxicological investigations. Aynur Abdulla et al. [42] developed a multi-channel microfluidic chip for long-term dynamic culture of brain organoids and precise regulation of flow rate, drug concentration, and exposure duration. Using this system, they modeled the chronic neurotoxicity of low-dose bisphenol S and assessed developmental and physiological responses, including proliferation, differentiation, and neural network activity, under varied exposure conditions. Continuous perfusion enhanced the uniformity of compound distribution and improved experimental reproducibility, providing an in vitro model closely aligned with in vivo exposure scenarios. Microfluidic platforms also play a crucial role in drug screening and personalized medicine by providing controlled, dynamic, and scalable culture environments that enable high-throughput evaluation of combinatorial treatments and mechanistic studies. Schuster et al. [12] demonstrated that microfluidic systems are particularly effective in recapitulating patient-specific treatment “time courses” in tumor organoid research, highlighting their critical role in modeling clinically relevant therapeutic regimens.

### 2.2 Matrix and scaffold engineering for organoid morphogenesis

In sensing-integrated organoid-on-a-chip systems, the matrix has transcended its role as a mere physical support to become a functional interface that determines the stability of the sensing-electrode junction and the signal-to-noise ratio of acquired data. Accordingly, engineered scaffolds with well-defined physicochemical benchmarks are essential to provide a reproducible background for real-time, dynamic, and quantitative on-chip studies. Decellularized extracellular matrix (ECM) hydrogels, while retaining tissue-specific biochemical cues, offer a more standardizable alternative by allowing researchers to tune the protein density and crosslinking degree, thereby optimizing the diffusion kinetics required for biosensors. For instance, kidney-derived ECM [43] and porcine intestinal ECM [44] have demonstrated the ability to maintain stem cell populations and promote mature vascularization more effectively than Matrigel. This structural fidelity is crucial for on-chip vascular perfusion monitoring, where the matrix's mechanical integrity prevents sensor drift caused by organoid-induced scaffold remodeling. Furthermore, by enriching the matrix with tissue-specific peptides, such as retinal pigment epithelium ECM proteins [45], researchers can markedly enhance synaptic marker expression and cellular responses to light stimulation. This biochemical optimization directly improves the functional fidelity of retinal organoids, ensuring that the electrophysiological or optical signals captured by on-chip sensors reflect a more mature and physiologically relevant neural activity.

Beyond ECM, protein-based hydrogels allow for the precise tuning of viscoelasticity and pore size. This structural control creates a stable mechanical interface and a defined conductive pathway, which are essential for enhancing the performance charge-transfer. Collagen-based matrices, integrated with the single-chain antibody scTS2/16 [46] or structured as mesoscale bundles [47], allow for clinical-scale organoid expansion and enhanced structural fidelity. This spatial organization directly correlates with the reliability of mechanosensing readouts by providing a physiologically relevant physical support that mimics native ECM bundles. Similarly, gelatin hydrogels, leveraging their matrix metalloproteinases (MMP)-responsive degradation and arginylglycylaspartic acid (RGD) adhesion motifs, enable high-precision regulation of the organoid microenvironment. Engineered gelatin platforms, such as host-guest supramolecular gelatin hydrogels [48] and enzyme-crosslinked matrices [49], provide dynamic mechanical cues and tunable stiffness that drive vascular maturation, such as transition to arterioles, and preserve tumor drug-response profiles. For on-chip integration, these well-defined hydrogel scaffolds maintain exceptional stability and reproducibility as a sensing medium throughout the organoid growth cycle. By precisely controlling the matrix degradation and swelling, researchers can ensure the organoids remain within the critical sensing distance, such as the electromagnetic “Debye length” for electrical signals or the precise optical focal plane for imaging, thereby minimizing signal drift and ensuring that real-time functional readouts are reproducible and reflect true physiological changes. Furthermore, the implementation of silk fibroin hydrogels (SF) introduces excellent mechanical robustness and programmable degradation profiles, essential for bone and cartilage organoid engineering on-chip. By employing microfluidic-assisted self-assembly to create RGD-SF-DNA microspheres [50], researchers can activate integrin-mediated signaling and stimulate glycosaminoglycan (GAG) biosynthesis, ultimately generating stable cartilage precursors with improved functional integrity. By minimizing the biological noise through such highly customized biochemical and mechanical niches, these engineered protein matrices stabilize the metabolic and electrophysiological baselines, thereby enhancing the sensitivity and reproducibility of real-time, dynamic functional maturation assays.

Furthermore, the integration of chemically defined synthetic and natural hybrid hydrogels enables the decoupling of mechanical stiffness from biochemical ligand density [51,52], a prerequisite for the quantitative modeling of sensor-detected “dose-response” relationships. Alginate hydrogels, for instance, utilize thiol-ene crosslinking [53] or encapsulation strategies [54] to act as defined physical boundary conditions. By attenuating pro-fibrotic mechanical signaling and preventing organoid fusion, alginate stabilizes organoid geometry and diffusion length scales, which is vital for impedance tomography and micro-electrode array (MEA) recording to avoid signal overlap from heterogeneous tissue clusters. Similarly, cationic chitosan-based composite capsules [55] leverage electrostatic interactions to regulate nutrient/metabolite exchange, while hyaluronic acid (HA) matrices [56] optimize tissue hydration and permeability. These modifications reduce central necrosis and ensure a stable sensing interface, facilitating high-fidelity long-term monitoring of photoreceptor maturation and neural lineage specification.

Beyond natural polymers, advanced synthetic platforms offer multi-modal metabolic and structural control. Hybrid polyethylene glycol (PEG)/HA hydrogels [57] and protease-sensitive PEG-4MAL systems [58] provide animal-free, reproducible microenvironments where stiffness, porosity, and cell-adhesive peptide densities are independently tuned. This level of control is further extended to the metabolic level through composite hydrogels like GelMA-PVA-TSPBA [59]. By incorporating reactive oxygen species (ROS)-scavenging functionality and biomimetic niches, these materials actively suppress oxidative stress and metabolic drift. For on-chip sensing, such microenvironmental regulation stabilizes the baseline of metabolic sensors, including pH and glucose probes, ensuring that the detected electrochemical signals reflect true physiological responses rather than matrix-induced artifacts. Taken together, these material strategies provide the reproducible physicochemical niche necessary for high-fidelity, multimodal on-chip monitoring across diverse organoid models, from spinal cord to bone marrow. For a clearer overview, the material properties and applications of the diverse scaffolding strategies are systematically summarized in Table 1.

**Table 1:** Engineering strategies and material logic of scaffolds for organoid-on-a-chip platforms.

Types	Key material properties	Targeted organoids	Impact on on-chip sensing	Ref
dECM	Features tissue-specific biochemical cues and tunable protein density; preserves the native ligand landscape to drive endothelial self-organization and vascularization.	Renal, Hepatic, Intestinal	Minimizes sensor drift; provides a biomimetic functional interface.	[[43], [44], [45]]
Collagen	Offers a low-immunogenicity fibrillar architecture; mimics native ECM bundles to provide stable 3D physical support and facilitate mechanosensing.	Patient-derived tumors; Intestinal	Improves signal-to-noise ratio (SNR) by enhancing structural fidelity.	[46,47]
Gelatin	Exhibits MMP-responsive degradability and thermo-reversibility; enables dynamic tuning of RGD adhesion sites and stiffness to drive arteriolar maturation.	Arteriolar-like patient-derived xenograft models	Ensures organoids remain within the critical sensing distance (such as focal plane).	[48,49]
Silk Fibroin	Provides exceptional mechanical robustness and programmable degradation; activates integrin-mediated signaling to stimulate GAG biosynthesis.	Bone and Cartilage	Maintains long-term baseline stability for mechanical/strain sensors.	[50]
Alginate	Characterized by bioinertness and rapid ionic crosslinking; acts as a physical boundary to prevent organoid fusion and decouple mechanical mechanosignaling.	Spinal cord; Anti-fibrotic kidney	Standardizes diffusion scales for impedance and MEA recording.	[53,54]
Chitosan/HA	Utilizes cationic interactions (Chitosan) or high hydration (HA) to regulate nutrient/cytokine exchange and mitigate central necrosis.	Liver; Retinal organoids	Optimizes permeability; reduces “biological noise” in metabolic readouts.	[55,56]
Synthetic hydrogel	Fully defined, animal-free matrices with independently tunable stiffness and ligand density; actively suppresses metabolic stress via ROS scavenging.	Spinal cord; Bone marrow	Stabilizes metabolic baselines (pH, glucose) by suppressing oxidative drift.	[[57], [58], [59]]

Crosslinking strategies in matrix engineering require a careful balance between structural stability and biological fidelity. High crosslinking density provides the mechanical robustness necessary for long-term on-chip cultivation [60]. This stability ensures that organoids remain within a fixed sensing distance, which minimizes signal drift in electrical and optical recordings [61,62]. However, excessive crosslinking can impair tissue viability by restricting the diffusion of nutrients and metabolic waste [63]. Such dense networks also directly affect the data output of integrated sensors by increasing the mass transfer resistance for soluble biomarkers. This resistance reduces the sensitivity and shortens the response time of electrochemical biosensors. Conversely, insufficient crosslinking leads to scaffold swelling or degradation, which causes fluctuations in the baseline of continuous metabolic monitoring. Furthermore, non-uniform crosslinking can induce shifts or refraction in optical signal transmission. Beyond these signal artifacts, the crosslinking degree dictates the matrix modulus and governs organoid phenotypes through cellular sensing of mechanical stimuli [64]. For instance, matrix stiffness modulates the transition of vascular organoids into arterioles and influences tumor drug response profiles [48]. Therefore, optimizing crosslinking degrees is essential to stabilize signal baselines and precision while maintaining the physiological relevance of the tissue model.

### 2.3 Physical and chemical stimulation to guide maturation

Beyond the regulation provided by microfluidic systems and culture matrices, recent studies have demonstrated that organoid development and function can be further modulated by external physical and chemical stimuli. These stimuli, such as electrical, magnetic, thermal, optical, and chemical cues, can recapitulate dynamic in vivo microenvironmental factors, thereby promoting cell differentiation, polarity establishment, and functional maturation.

#### 2.3.1 Physical stimulation

**Fig. 2:**
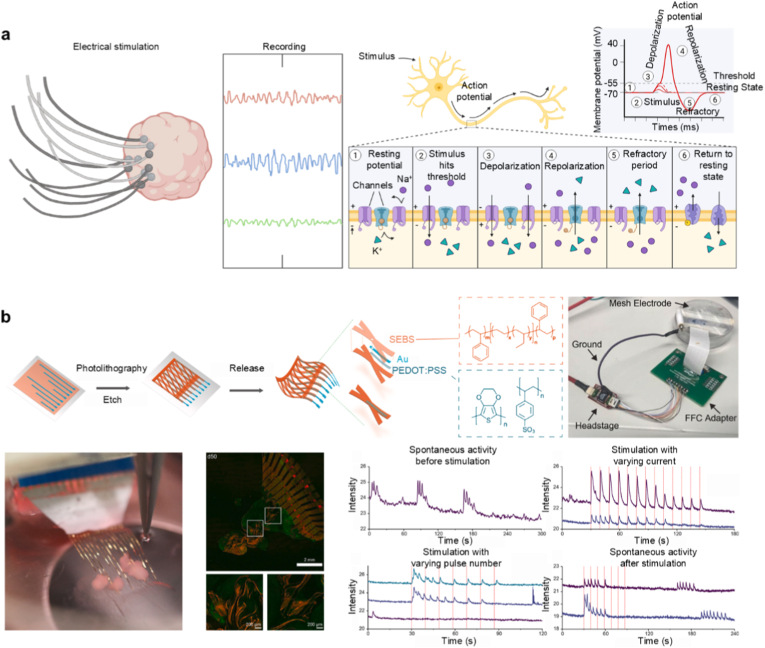
Electrical stimulation technologies for modulating bioelectric states in organoid-on-a-chip systems. (a) Mechanisms of bioelectric state modulation via electrical stimulation, illustrating the sequential opening and closing of transmembrane ion channels across different electrophysiological stages; (b) Induction and capture of high-SNR neural/cardiac electrophysiological signals using flexible mesh microelectronics, showing the fabrication pipeline and high-SNR neural/cardiac electrophysiological recording under varying stimulation patterns [65], copyright 2022, Elsevier, license: 6285501111663.

Electrical stimulation is uniquely suited for on-chip integration as it provides a high-speed, direct handle to modulate bioelectric states (Fig. 2a). Stretchable mesh microelectronics [65] leverage a flexible design to maintain a stable bio-interface, which minimizes impedance drift and interfacial noise during long-term stimulation (Fig. 2b). This tight electrical coupling ensures that evoked intensity-dependent Ca^2+^ responses and synchronized neural activities are captured with high signal-to-noise ratios. By providing a controlled electrical perturbation, these systems enable sensors to benchmark organoid functional maturity against predictable firing patterns, offering higher diagnostic fidelity than the observation of stochastic spontaneous activity. Furthermore, the use of electrobio-reactors [66] to apply localized electric fields has been shown to accelerate organoid development via the PI3K/Akt pathway, effectively shortening the “waiting period” for sensors to detect mature, baseline-stable electrophysiological signals. Similarly, in non-neural models [67], physiological-strength electric fields modulate ionic flux and lumen expansion, providing a controllable biophysical input that allows sensors to benchmark tissue tension and barrier maturation against a known electrical perturbation. The translational scope of active electro-biophysical cues has recently been extended to direct the maturation of highly specialized sensory mechanoreceptors. Hu et al. [68] engineered a novel biohybrid cochlea-on-a-chip platform that integrates a highly conductive polypyrrole-polydopamine (PDA)-Matrigel hydrogel matrix. Benefiting from this setup, the superior electrical conductivity of the substrate synergizes with artificial cochlear implant electroacoustic stimulation to promote intercellular electrical signaling and activate voltage-gated calcium channels, thereby triggering intracellular Ca^2+^ transients to drive the proliferation and expansion of inner ear progenitor cells. Controlled by this electro-responsive microfluidic device, the encapsulated progenitors successfully undergo spontaneous self-organization and lineage differentiation into structurally mature, high-viability 3D cochlear organoids equipped with functional hair cells expressing specific mechanosensitive markers, establishing a robust in vitro biological model of complex inner ear microstructures.

**Fig. 3:**
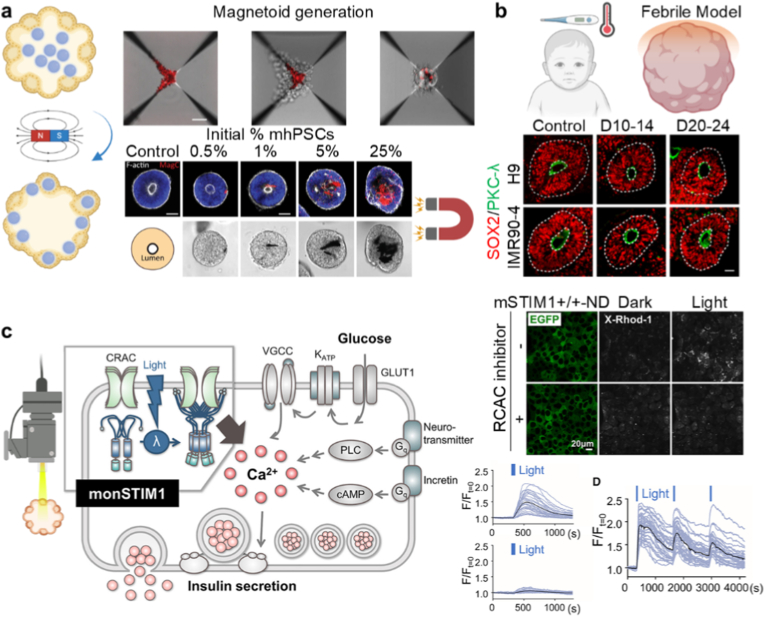
Modulation of biological responses in organoids via magnetic, thermal, and optical physical stimuli. (a) Magnetic nanoparticle-induced “Magnetoids” generation and the regulatory effect of varying mhPSC percentages on organoid structuring [69], published under CC BY license; (b) Periodic thermal stimulation model mimicking prenatal fever, showing the spatial expression of developmental biomarkers (SOX2/PKC-λ) across distinct timelines [70], published under CC BY license; (c) Real-time optical control of insulin secretion in islet organoids based on optogenetic technology, demonstrating Ca^2+^ influx and time-series fluorescence intensity profiles [71], published under CC BY-NC-ND license.

Magnetic, thermal, and optical cues provide unique, non-invasive routes to deliver localized stimuli and drive spatiotemporal biological responses. Magnetic actuation offers a wireless method to deliver mechanical forces. Abdel Fattah et al. [69] embedded magnetic nanoparticles into organoids to create magnetic clusters that exert localized mechanical forces. Under an external magnetic field, these clusters drive cytoskeletal remodeling and long-term structural changes, promoting asymmetric tissue growth and proliferation in neural organoids. For on-chip sensing, such patterned growth enables the detection of spatially non-uniform mechanical responses that would be difficult to resolve under conventional macro-scale stretching (Fig. 3a). Xu et al. [70] established a model of periodic thermal stimulation in human brain organoids to mimic prenatal heat exposure. Cyclical heating was found to shift the proliferation-differentiation balance by reducing neural progenitor numbers while increasing neuronal differentiation, a process mediated by aberrant WNT signaling gradients. Monitoring these thermally-induced molecular and structural malformations on-chip provides a platform for investigating how environmental change influences neurodevelopmental stability (Fig. 3b). Finally, optical stimulation offers high spatiotemporal precision without physical contact. Jieun Choi et al. [71] integrated the light-sensitive monSTIM1 into pancreatic islet-like organoids, enabling light-triggered intracellular Ca^2+^ transients. This optical control allowed for the reversible modulation of β-cell insulin secretion in real-time. By utilizing light-based activation, researchers can drive specific functional programs, such as C-peptide production in patient-derived organoids, which offering a highly controllable input to validate the dynamic response and sensitivity of integrated biochemical sensors. Taken together, these stimulation modalities offer versatile ways to actively perturb organoid structure and function, providing the necessary biological inputs for real-time monitoring platforms to track the dynamic functional trajectory of organoids on-chip (Fig. 3c).

#### 2.3.2 Chemical stimulation

**Fig. 4:**
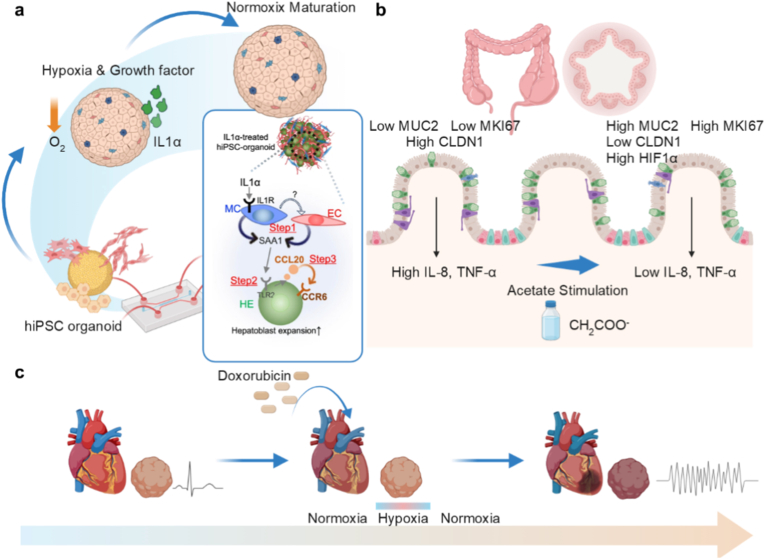
Applications of chemical stimuli in organoid morphogenesis and functional regulation. (a) Simulation of liver development stages via hypoxia induction combined with placental factor stimulation, illustrating the cell-cell interaction pathways driving hepatoblast expansion [72], copyright 2023, Springer Nature, license: 6285520751476; (b) Enhancement of barrier function in ulcerative colitis organoids by acetate stimulation, showing the modulation of barrier markers and the concurrent downregulation of inflammatory cytokines; (c) Simulation of myocardial infarction and drug-induced cardiotoxicity using doxorubicin concentration gradients.

Unlike physical cues that act primarily through fields, chemical stimulation offers programmable, spatiotemporal control over signaling and exposure history, closely mirroring developmental gradients and disease-relevant dosing dynamics. This approach is fundamental for regulating organoid morphogenesis and functional maturation, with growth factor-mediated modulation representing a premier strategy to recapitulate in vivo developmental signals. Recent studies have demonstrated that incorporating developmentally inspired cues can significantly enhance the structural and functional fidelity of organoids. For instance, Yoshiki Kuse et al. [72] utilized a “hypoxia induction plus placental factor, such as IL1α, stimulation” approach in iPSC-derived hepatic organoids to mimic embryonic developmental stages (Fig. 4a). This sequential modulation promoted progenitor expansion and increased metabolic complexity, successfully recapitulating key physiological transitions and improving the transplant potential of the resulting tissues. In tumor modeling, the precision of chemical cues is equally vital for preserving genotypic and phenotypic stability. Tan et al. [73] proposed a “low growth factor culture system” using essential regulators, including FGF10, A83-01, and SB202190, to attenuate exogenous signal dependence. By modulating signaling intensity, this system preserves tumor heterogeneity and genetic stability, thereby enhancing the predictive value of the model for drug responses. These advancements in chemical-mediated modeling provide the physiologically relevant biological substrates necessary for reliable downstream functional analysis and real-time monitoring on-chip.

Chemical stimulation can further precisely regulate cellular behavior and tissue function by modulating ion concentrations or drug exposure. Salt concentration, as a chemical cue, has been instrumental in investigating epithelial barrier integrity and inflammatory responses. In colonic organoid monolayers derived from ulcerative colitis patients, high acetate concentrations [74] significantly enhanced epithelial barrier function, as quantitatively evidenced by increased transepithelial electrical resistance (TEER), as shown in Fig. 4b. This modulation is accompanied by the upregulation of HIF1α, MUC2, and MKI67, alongside a reduction in pro-inflammatory cytokines IL8 and TNFα, demonstrating that controllable chemical milieus can activate endogenous protective pathways for inflammatory bowel disease (IBD) drug screening. Similarly, drug concentration gradients exert significant effects on the organoid microenvironment and functional stability. Dylan J. Richards et al. [75] employed human cardiac organoids to model myocardial infarction and drug-induced cardiotoxicity by modulating doxorubicin concentrations and oxygen gradients (Fig. 4c). This spatial control allowed the organoids to mimic functional heterogeneity across infarct, border, and remote zones, mirroring regionalized injury patterns. High drug concentrations, particularly under hypoxic conditions, induced dose-dependent cardiotoxic effects, including decreased contractility, arrhythmias, and pronounced calcium handling defects. By inducing these non-genetic pathological states, chemical modulation provides a robust platform for investigating drug toxicity and hypoxia-induced mechanisms, ensuring that captured physiological readouts reflect complex tissue-level responses. To provide a clearer delineation of these multi-modal microenvironmental effects, the distinctive regulatory parameters of external biophysical forces and biochemical pathways are systematically decoupled and summarized in Table 2. Specifically, this framework bridges physical executing routes, such as fluid kinetics and matrix rigidity, with chemical inputs like developmental factors and dosing, explicitly highlighting their functional impacts on integrated on-chip sensing.

**Table 2:** Overview of external biophysical and biochemical microenvironmental signals and their functional impacts on sensing-integrated organoid-on-a-chip platforms.

External signal classification	Input parameters and stimuli	Structural execution route	Functional impact on on-chip sensing and signal acquisition	Ref
Biophysical axis	Hydrodynamic perfusion flow	Hydrodynamic trapping and continuous fluidic refreshment loops.	Maximizes nutrient/oxygen homogeneity; prevents localized waste accumulation to stabilize baseline metabolic tracking.	[37]
	Fluid shear stress	Active mechanical loading driven by steady fluid flow kinetics inside chips.	Standardizes tissue diffusion length scales and physical boundaries, critical for reliable electrical/impedance recording.	[38]
	Cyclic medium flow	Chronological oscillatory medium flow loops across bECM scaffolds.	Minimizes sample-to-sample volumetric variance, delivering stable and reproducible MEA lines.	[39]
	Numerical CFD-fluid-structure interaction (FSI) WSS modalities	Patient-specific computed tomography angiography (CTA) data-driven computational hemodynamics with microfluidic execution.	Yields quantitative, boundary-defined mechanical vector values to precisely calibrate integrated cell strain/tension sensors.	[40]
	Perfusable vascular interfaces	Multicellular co-culture integration within shear-perfused microfluidic channels.	Reduces physical tissue motion artifacts; ensures high-fidelity, contrast-clear feeds for live image segmentation.	[41]
	Tissue-specific matrix stiffness	Regulating native protein matrix densities to resist cellular traction forces.	Prevents long-term sensor drift and physical structural misalignment caused by organoid-induced cell-matrix remodeling cascades.	[[43], [44], [45]]
	Viscoelasticity and modulus tuning	Structuring mesoscale fiber bundles, supramolecular or enzyme-crosslinked nets.	Anchors organoids tightly within the sensor's critical sensing distance to avoid signal artifacts.	[[47], [48], [49]]
	Physical crosslinking boundaries	Utilizing rapid ionic crosslinking or encapsulation strategies to form well-defined physical boundary conditions.	Decouples mechanical signaling pathways and prevents unconstrained tissue fusion, standardizing diffusion scales to avoid signal overlap in impedance tomography and MEA recording.	[53,54]
	Mechanical field actuation	Targeted mechanical loading of tissue via externally controlled wireless magnetic fields.	Facilitates the high-resolution tracking of spatially non-uniform mechanical strain and directional structural responses.	[69]
	Cyclical thermal perturbations	Executing automated, chronological febrile-range thermal culture schedules inside incubators.	Supplies a reliable, controllable in situ physical stress test framework to monitor neurodevelopmental stability.	[70]
	Physiological electrical fields	Deploying flexible stretchable mesh electronics or localized active electric fields.	Synchronizes stochastic cellular firing into predictable baseline patterns, minimizing interfacial noise to maximize SNR.	[[65], [66], [67], [68]]
	Targeted receptor photostimulation	Direct spatiotemporal laser/optical stimulation to trigger intracellular Ca^2+^ cascades without contact.	Delivers completely non-invasive, programmable biochemical inputs to validate the dynamic response thresholds of biosensors.	[71]
Biochemical axis	Oxygen dynamics and hypoxia flux	Active microfluidic control of gas-permeable fluidic channels and flowing flux.	Calibrates microenvironmental gaseous boundaries; prevents sensor saturation and baseline oxidative drift.	[36]
	Sequential developmental signaling	Programmatic delivery of embryonic sequential developmental signaling and hypoxia cues.	Eliminates developmental “biological noise” and metabolic fluctuations during long-term screening baseline setup.	[72]
	Exogenous niche regulation	Culturing PDOs with essential core regulators to lower signal reliance.	Establishes high-fidelity biological substrates necessary for stable downstream pharmacodynamic data validation.	[73]
	Dynamic metabolite gradients	Programmatic microfluidic orchestration of localized specific metabolite fractions.	Provides stable chemical validation inputs to benchmark on-chip TEER sensors.	[74]
	Chemical toxicity dosing	Multi-channel automated fluidic routing for precise concentration and chronic duration tracking.	Standardizes analytical detection limit (LOD) and inputs for long-term downstream secretome analytics.	[42]
	Compartmentalized drug gradients	Establishing localized chemical concentrations and spatial oxygen boundaries across tissue zones.	Induces predictable, regionalized pathological states, allowing sensors to track complex tissue-level calcium handling defects.	[75]
	Interfacial epitope optimization	Enriching structural hydrogels with tissue-specific sequence peptides or cell-adhesive RGD motifs, or silk fibroin microstructures.	Boosts functional tissue differentiation fidelity, ensuring that captured electrophysiological/optical signals reflect true states.	[45,46,50]
	Electrostatic charge interactivity	Utilizing cationic polymer charges and highly hydrophilic glycosaminoglycan architectures.	Optimizes interface permeability and mass transfer; reduces biological noise in metabolic readouts by mitigating central necrosis.	[[55], [56], [57]]
	Active metabolic stabilization	Chemical covalent integration of active ROS-scavenging phenylboronic acid functional groups.	Actively suppresses cellular metabolic drift, protecting electrochemical pH/glucose probes from matrix-induced chemical artifacts.	[59]

## 3 Real-time and in situ monitoring technologies in organoid-on-a-chip

The integration of real-time and in situ monitoring technologies is fundamental to transforming organoid-on-a-chip platforms from simple culture vessels into sophisticated analytical tools for biomedical research. By transcending the limitations of conventional destructive endpoint assays, these integrated sensing modalities enable the continuous capture of dynamic biological information across multiple dimensions, spanning from metabolic biochemical fluctuations and bioelectric activity to biomechanical dynamics. The synergy between advanced functional materials and miniaturized sensing hardware provides unprecedented temporal resolution, allowing for a non-invasive and high-fidelity understanding of organoid development, disease progression, and therapeutic responses.

### 3.1 Electrical monitoring

Electrical monitoring in organoid-on-a-chip systems broadly encompasses electrochemical sensing of biochemical analytes and electrophysiological recording of cellular electrical activity. While electrochemical sensing converts biochemical events, such as redox reactions or affinity capture, into measurable current or voltage signals, electrophysiology captures endogenous bioelectric activity arising from ion-channel dynamics and intercellular coupling. These complementary modalities provide essential tools for in situ, real-time characterization of organoid functional status, metabolic activity, and drug responses, overcoming the limitations of traditional endpoint assays like ELISA.

Electrochemical sensors have emerged as vital components for monitoring soluble biomarkers due to their high sensitivity and ease of miniaturization. Shin et al. [76] pioneered a label-free microfluidic electrochemical biosensor integrated with a human liver-on-a-chip, achieving long-term monitoring of albumin (ALB) with a LOD of 0.09 ng/mL and a sensitivity of 1.35 (log (ng/mL))^−1^. To conquer the microenvironmental boundaries of sensor saturation and biofouling, this platform heterogeneously integrated pneumatic polydimethylsiloxane (PDMS) valves with on-chip gold microelectrodes modified by self-assembled monolayers and antibody affinity matrices. Directed by automated fluidic routing, this material configuration precisely delivered micro-samples and mild elution matrices under minimal dynamic shear stress. This microfluidic-surface chemistry coupling successfully prevented biofouling and interfacial disturbances without damaging the active catalytic layers, maintaining baseline signal drift within 5% across dozens of automated binding-regeneration cycles. To bridge the gap between structural complexity and high-throughput multi-variable chemical screening, advanced platforms must transcend rigid planar configurations toward structure-functional material integration. Brooks et al. [77] engineered an integrated 3D-printed electrochemical multi-well plate utilizing carbon black/poly-lactic acid (CB/PLA) conductive composites to transcend traditional planar electrode configurations. Within the insulating PLA matrix, the 3D interconnected percolation topology formed by conductive carbon black nanoparticles is rich in carbon-based defect sites and electroactive edge planes. This architecture facilitates an exceptional heterogeneous electron transfer rate toward electroactive small molecules, thereby lowering the oxidation potential of serotonin. Through the arrayed multi-well boundaries, the platform simultaneously achieves physical isolation and parallel sensing across independent cellular microenvironments. This setup successfully tracked the real-time serotonin secretion from multiple groups of intestinal enterochromaffin cells within a linear range of 0.1-2 μM with a sensitivity of 13.1 nA/μM, thereby overcoming the traditional trade-off between tissue complexity and high-throughput screening. In kidney organoid differentiation, Suhito et al. [78] utilized label-free electro-signatures to monitor cellular composition, seamlessly balancing the trade-off between cell adhesion and mass transport resistance at the electrode interface. By limiting the hydrogel thickness, they minimized the diffusion barrier for extracellular metabolites, allowing rapid mass transport to the electrode surface to facilitate efficient faradaic redox reactions. A precisely controlled matrix thickness prevents the excessive accumulation of non-conducting hydrogel networks that would otherwise introduce substantial diffusion resistance, drastically increasing the interface impedance and attenuating transient redox currents into background electronic noise. Tailoring the structural alignment of such thin-film hydrogels through Young's modulus adaptation, biocompatibility optimization, and intrinsic conductivity refinement effectively suppresses baseline oxidative drift while guaranteeing robust organoid construction, thereby preventing abrupt signal errors throughout long-term tracking. Consequently, highly proliferative, undifferentiated hiPSCs and off-target stromal cells trigger distinct electron transitions at near 0 V potentials due to their hyper-metabolic profiles; conversely, successfully differentiated renal-specific cells generate unique redox signals at approximately 0.3 V proportional to cell abundance and tubular maturation. This non-destructive method achieved an hiPSC LOD of 21,363 cells, replacing traditional destructive endpoint assays with real-time lineage specification tracking. Furthermore, to address the challenges of monitoring intratumor heterogeneity inaccessible by conventional 2D cultures, Nashimoto et al. [79] employed scanning electrochemical microscopy (SECM) for the non-invasive assessment of oxygen metabolism within single tumor organoids as small as 100 μm in diameter. By precisely regulating the electrochemical polarization potential of the UME material, the local oxygen reduction reaction is strictly confined to a four-electron pathway that yields entirely benign H_2_O, fundamentally eliminating cytotoxicity or physical artifacts induced by ROS or other radical-driven side reactions. Because highly proliferative tumor sub-populations (L-cell lineages) and slow-growing, drug-resistant dormant sub-populations (S-cell lineages) are heterogeneously intertwined, the oxygen flux leaking from the organoid physical boundary exhibits highly anisotropic spatial gradients. SECM captures these spatial fluctuations by recording local reduction current decay within the diffusion boundary layer, thereby accurately quantifying the oxygen consumption rate and mapping the metabolic heterogeneity with high spatial fidelity.

In neural research, the dynamic release of neurotransmitters serves as a benchmark for neuronal maturation, yet in situ detection remains obstructed by ultra-low biological abundances and rapid molecular diffusion. Nasr et al. [80] developed enzyme-modified capillary microelectrodes utilizing self-organized nano-structural modifications. This matrix modification significantly expanded the electrochemically active surface area to accelerate enzymatic electron tunneling, achieving sensitive in situ glutamate monitoring with an LOD of 5.6 μM. The nanoscale interface engineering drastically lowers the interface impedance and promotes robust noise reduction by facilitating rapid electron-transfer ability at the neural boundary. For non-electroactive macromolecular biomarkers implicated in neurodegenerative disorders, electrochemical platforms must resolve the low abundance of target signaling species within complex culture matrices. To establish a highly specific diagnostic boundary, Lee et al. [81] designed electrochemical sensor leveraging an epitope-molecularly imprinted conducting polymer. This platform utilizes synthesized α-synuclein peptide epitopes as templates for the in situ electropolymerization of a conductive poly (aniline-co-m-aminobenzenesulfonic acid) matrix. The spatial recognition and imprinting efficiency are driven by the localized affinities between the free C-terminal -COOH of the target peptides and the complementary functional amine groups of the monomers. This tailored polymer topology drastically optimizes the binding capacity and electronic sensitivity of the sensor, enabling biomimetic polymer cavities to achieve the non-destructive dynamic analysis of α-synuclein at levels as low as 10 fg/mL. The high conductivity of the polyaniline derivative minimizes charge-transfer resistance at the bio-interface to effectively suppress background electronic fluctuations, while its optimized biocompatibility actively resists non-specific protein fouling to safeguard high-quality signal outputs. Tailoring the polymeric matrix structure to comply with the mechanical boundaries of ultra-soft neural tissues circumvents the Young's modulus mismatch, preventing microscale delamination and mechanical damage at the electrode surface to maintain baseline fidelity throughout long-term tracking. Furthermore, to resolve highly transient exocytotic release, such as vesicular dopamine flux in midbrain organoids, sensors must possess exceptional spatial and temporal resolutions to capture stochastic currents within confined sub-cellular micro-domains. To analyze vesicular storage in midbrain organoids, Zhu et al. [82] developed intracellular vesicle impact electrochemical cytometry utilizing a customized conical carbon-fiber nano-tip microelectrode to non-destructively pierce individual living dopaminergic neurons. The physical chemistry core of this platform relies on the in situ adsorption and electro-fusion of single synaptic vesicles onto the nano-electrode surface, allowing the recording of transient faradaic currents dictated by fusion pore dynamics. This nano-interface successfully quantified a significant chemical storage deficiency in young-onset Parkinson's disease models, resolving approximately 15.22 × 10^4^ dopamine molecules per vesicle compared to 27.64 × 10^4^ in healthy controls. More recently, Wang et al. [83]developed an non-destructive real-time platform based on a field-effect transistor (FET) biosensor platform featuring a multi-compartmental material design that integrates a transwell interaction chamber, a transmission microchannel, and a liquid-gate graphene FET zone. This architecture guides drug-stimulated secretions from 3D liver organoids directly downstream. Benefiting from the high carrier mobility of the graphene channel, the specific capture of negatively charged ALB molecules repels internal electrons, introducing a localized potential variance. The charge-screening mechanism successfully translates dynamic biomarker fluctuations into high-fidelity transistor current outputs for continuous, real-time monitoring, achieving a LOD of 1 pg/mL with a robust electrical potential shift exceeding the threefold noise threshold.

Electrophysiological monitoring is critical for assessing cellular function and network interactions in excitable tissues. In cardiac research, Tirgar et al. [84] utilized liquid metal electrode arrays to monitor calcium transients at the single-cell level, analyzing parameters such as time-to-peak and decay times (Decay 70/50/30). The fundamental material and optical logic of this platform rely on the structural customization of a gradient-index lens to extend the working distance, which utilizes non-invasive optical interrogation rather than rigid planar electrodes. This configuration effectively circumvents the core restriction of near-zero working distances inherent in conventional microscopes, thereby establishes a favorable operational boundary between optical sensor and the microfluidic chip matrix. Utilizing a liquid-metal-based architecture natively addresses the extreme Young's modulus mismatch against dynamically beating cardiac tissues. Furthermore, the high intrinsic conductivity and fluidic deformability of liquid metals significantly lower interface impedance and suppress motion-induced background noise during continuous rhythmic contractions, seamlessly coupling this compliant electronic interface with precise matrix configuration to acquire long-term, high-fidelity signals. To overcome the spatial limitations of fixed electrodes, Jacques et al. [85] developed a platform combining photoelectrochemical imaging with light-addressable voltage sensors. By using focused light as “virtual electrodes” on semiconductor surfaces, this system enables non-invasive, high-sensitivity monitoring of single-cell action potentials and intercellular coupling within 3D organoids. This light-addressable architecture reveals intricate network synchronization dynamics and provides high-precision data for pharmacological profiling, accurately resolving the effects of compounds like verapamil and blebbistatin on organoid electrophysiology. Similar advancements have been applied to ocular and neural tissues. The fundamental material logic of this platform relies on a self-assembled monolayer-modified silicon-on-sapphire substrate, utilizing its narrow bandgap and high charge-carrier mobility for high-speed potentiometric sensing. This configuration effectively prevents the electrolyte gap in physiological media from screening the cell's membrane potential. Crucially, applying a controlled force creates a stable contact between the organoid and the sensor, eliminating movement artifacts for accurate action potential tracking. To accommodate 3D architectures, Choi et al. [86] introduced shape-adaptive, organoid-encapsulating shell MEAs that ensure close conformal contact with cardiac organoid surfaces. This 3D electrode network overcomes the spatial limitations of traditional 2D planar MEAs. Notably, the study demonstrated that calculating action latency via the slope method (0.875 accuracy) significantly outperforms the traditional amplitude-based method (0.696) due to its superior resistance to signal attenuation. The conformal 3D wrapping establishes a stable bioelectronic interface around the tissue, preventing signal loss to ensure high-fidelity, spatiotemporal mapping of organoid synchronization. Electrophysiological monitoring of ocular tissues can similarly be applied to retinal organoids. Lee et al. [87] developed 3D-printed liquid metal microelectrodes for in situ monitoring of retinal ganglion cell development. To enhance signal fidelity, the electrodes were modified with Pt nanoclusters, which significantly reduced interfacial impedance from 506.93 kΩ to 114.36 kΩ. This modification minimized noise, enabling high-resolution recording of action potentials and synaptic activity within early-stage retinal organoids. The flexible and adaptive nature of these electrodes ensures close contact with retinal layers while preserving tissue integrity, providing a robust platform for studying neural network formation and drug responses.

For brain organoids, capturing intricate 3D network activity requires high-precision, multi-channel interfaces. Kim et al. [88] developed a magnetically reconfigurable 3D MEA using Ga-In liquid metal. By employing a 6-axis magnetic tilt system with 0.002° resolution, the electrodes can be precisely reoriented to achieve a maximum displacement of 234 μm, increasing the detectable area by approximately 891 times. The material design logic utilizes microscale EGaIn pillars matching the tissue's Young's modulus ensures reliable contact with diverse neuronal groups. By combining a ferromagnetic coating with liquid-metal deformability, this magneto-fluidic coupling enables the active tracking of neural networks by allowing single sites to dynamically scan multiple coordinates, overcoming the spatial constraints of static arrays without destroying the 3D cytoarchitecture. Similarly, Yang et al. [89] introduced the kirigami electronics (KiriE) platform, which transforms 2D flexible devices into 3D basket-like structures to envelop suspended organoids. Integrated with DGCR8 cortical organoids, the KiriE system successfully captured disease-related phenotypes of 22q11.2 deletion syndrome, revealing spontaneous firing rates three times higher than controls (with peak rates reaching 7 Hz). The structural principle relies on a kirigami-folded, open-mesh basket that seamlessly encloses intact organoids in suspension, thereby avoiding the developmental and structural disruption caused by traditional substrate-attached alternatives.

Collectively, electrochemical and electrophysiological monitoring platforms provide complementary, multi-scale insights, revealing clear trade-offs between materials and performance. Electrochemical sensors excel at tracking dynamic soluble secretomes and metabolic fluxes, whereas electrophysiological arrays resolve millisecond-scale electrical networks. For high-throughput screening, electrochemical platforms offer excellent scalability but suffer from progressive sensor signal drift and baseline fluctuations caused by non-specific protein adsorption in complex biological media. Conversely, shape-adaptive or reconfigurable MEAs deliver exceptional spatial fidelity for excitable circuits but remain ineffective for non-excitable metabolic tissue models like liver or pancreas organoids. Although high-surface-area nanoparticles, such as carbon black or platinum nanoclusters, minimize interface impedance, they face severe mechanical instability and bio-interfacial challenges in long-term 3D cultures. Hydrogel swelling and organoid creep frequently induce microscale delamination of nanoparticles from conductive substrates, causing impedance fluctuations and risks of leaching-induced cytotoxicity. Furthermore, this elevated surface area inherently accelerates non-specific protein adsorption, causing severe sensor signal drift. Beyond surface fouling, enforcing tight contact to suppress motion artifacts introduces severe structural perturbations; the physical mismatch with the ultra-soft embryonic Young's modulus of organoids inflicts excessive compression that restricts tissue expansion and alters native phenotypes via mechanosensitive pathways. Consequently, engineering mechanically compliant interfaces and customizing active anti-biofouling architectures, such as the integration of zwitterionic coatings or PEGylation, remain paramount to balance cellular biocompatibility with continuous signal longevity.

### 3.2 Optical monitoring

Spectroscopic techniques are increasingly recognized as key analytical tools for in situ, label-free, and real-time monitoring of organoid systems. As highlighted by Wei Mao et al. [90], spectroscopic approaches, including fluorescence, Raman, and infrared spectroscopy, enable continuous tracking of biochemical and biophysical changes during stem cell proliferation and organoid differentiation within 3D microenvironments. By providing high spatiotemporal resolution, these methods offer dynamic insights into tissue development and drug responses while preserving organoid structure and viability, thereby enhancing the functional depth and translational relevance of organoid platforms in drug screening and regenerative medicine.

Optical monitoring strategies, encompassing spectroscopic analysis and fluorescence imaging, provide non-invasive, high-resolution insights into the biochemical and functional trajectories of organoids. Raman spectroscopy, in particular, offers a label-free “fingerprinting” capability to resolve molecular compositions. Tubbesing et al. [91] developed an in-situ characterization method based on confocal Raman spectroscopy for 3D salivary gland organoids. By acquiring comprehensive Raman “fingerprints” of fully hydrated organoids, the approach captures protein, lipid, and nucleic acid vibrational signals, such as amide I (∼1650 cm^−1^), amide III (∼1250 cm^−1^), and DNA phosphate backbone (∼785 cm^−1^), while preserving cell-matrix interactions. These spectral features exhibited significant variations under EGF- and FGF2-induced differentiation states, serving as reliable phenotypic markers. The optical logic leverages inelastic light scattering for label-free profiling, bypassing the severe water interference of infrared spectroscopy in hydrated tissues. While optimizing confocal settings helps isolate intracellular vibrational signatures from the surrounding ECM, the biophysical and chemical attributes of the scaffolding matrix remain equally vital for long-term fidelity. Adapting the matrix's Young's modulus to achieve robust three-dimensional structural stability prevents the organoid from drifting out of the confocal focal plane due to tissue deformation or creep, thereby eliminating optical motion artifacts. Concurrently, optimizing the chemical purity and biocompatibility of the matrix actively suppresses non-specific autofluorescence, fundamentally minimizing background noise to safeguard baseline spectral stability throughout extended tracking. Similarly, Pettinato et al. [92] integrated micropatterned microwell arrays with Raman and confocal light-scattering spectroscopy to enable in situ monitoring of hiPSC-derived liver organoids. This platform permitted real-time, label-free tracking of spectral changes associated with DNA (∼785 cm^−1^), proteins (amide I, ∼1650 cm^−1^), and lipids (C-H stretching, ∼2850–2950 cm^−1^), revealing dynamic trajectories of chromatin remodeling and metabolic reprogramming during differentiation. This platform utilizes cell-repellent microwell arrays to rapidly aggregate uniform organoids without a necrotic core. By combining Raman and confocal light-scattering spectroscopy, the system fully leverages intrinsic optical contrast to track both metabolic states and chromatin structures without exogenous labels, perfectly preserving the transplantation safety of the organoids. To handle the complexity of neural systems, Bruno et al. [93] combined Raman spectroscopy with machine learning to enable label-free, real-time monitoring of cortical organoid biochemical remodeling. Using optically transparent material platforms for deep laser penetration, the study captured nucleic acid (∼785 cm^−1^), protein (∼1650 cm^−1^), and lipid (∼2850–2950 cm^−1^) signals without disrupting organoid development. By integrating principal component analysis, support vector machine, and random forest algorithms with Raman spectroscopy, this platform replaces manual peak-picking with automated chemometric recognition. The bias-free approach successfully identifies characteristic fingerprints of glycan and lipid metabolism, enabling the quantitative tracking of cortical maturation across differentiation stages. Spectroscopic monitoring also demonstrates significant utility in drug response assessment. Kothadiya et al. [94] illustrated the potential of Raman spectroscopy for in situ, real-time evaluation of chemotherapeutic effects in multi-layered 3D models. To couple with this complex material environment, depth-resolved Raman spectroscopy drives incident photons into the scattering matrix to capture localized inelastic signals without physical disruption. Following cisplatin treatment, spectral features associated with DNA backbone vibrations (∼783 cm^−1^), amino acid C-H bending (∼1338 cm^−1^), and amide III (∼1248 cm^−1^) exhibited depth- and time-dependent changes, reflecting metabolic heterogeneity at the molecular level.

Beyond conventional spectroscopic approaches, fluorescence-based techniques offer distinct advantages in active emission, high spatial localization, and quantitative real-time monitoring, making them pivotal for the in situ characterization of organoid dynamics. To address the challenge of limited multiplexing in 3D tissues, Reynolds et al. [95] developed a multi-cycle imaging platform for patient-derived glioblastoma organoids based on bioorthogonal click chemistry. The platform relies on the ultrafast kinetics of the bioorthogonal tetrazine/trans-cyclooctene reaction to systematically quench fluorescent signals between successive imaging rounds. It is of paramount importance to note that the bioorthogonal approach establishes a non-invasive chemical-optical interface within the living 3D microenvironment, replacing traditional destructive fixation with continuous tracking of diverse markers, including CD133, HER2, EGFR, and Vimentin, to map tumor spatial heterogeneity.

The development of specialized fluorescent probes further extends monitoring capabilities to organoid-level physiological functions. For instance, Zhang et al. [96] introduced Lyso-BFP, a pH-responsive probe for the in situ monitoring of lysosomal acidification. This probe operates via a photo-induced electron transfer (PET) mechanism with excitation at 405–450 nm and emission at 460–500 nm, where protonation of the hydroxyethyl piperazine moiety under acidic conditions inhibits PET to activate an off-on fluorescent switch. To interface seamlessly with the living microenvironment, a salicylamide group optimizes the probe's pK_a_ and water solubility, establishing a non-fluorescent buffer pool within the cytoplasm. When intra-organelle probes photobleach during long-term tracking, inactive molecules from this cytoplasmic reservoir continuously diffuse across the biological boundary to replenish the fluorophores, enabling wash-free, non-invasive imaging of organoid microenvironments. Similarly, to track ion dynamics within thick 3D structures, Müller et al. [97] reported a deep-red fluorescent potassium sensor based on a crown-ether-functionalized BODIPY fluoroionophore encapsulated within cationic Eudragit RL100 polymer nanoparticles. From a materials perspective, the structural definition of this polymeric nanoparticle matrix directly dictates noise reduction and long-term signal stability for high-precision optical imaging. Optimizing the surface biocompatibility of the Eudragit matrix minimizes the formation of a protein corona, which would otherwise alter nanoparticle surface charges, induce non-specific biological clustering, and introduce substantial background fluorescence noise. Furthermore, the stable encapsulation provided by the dense polymer matrix isolates the core fluoroionophore from environmental enzymatic degradation and premature dye leakage, effectively suppressing baseline fluorescence drift to guarantee high-fidelity signal output during extended spatiotemporal tracking. By operating in the deep-red spectrum with excitation at ∼630 nm and emission at 650–680 nm, and pairing with fluorescence lifetime imaging microscopy, the platform minimizes background interference and enhances tissue penetration, enabling the high-precision mapping of spatiotemporal K^+^ distributions when combined with fluorescence lifetime imaging. To eliminate the need for exogenous probes and ensure long-term monitoring, genetically encoded reporters have been integrated into organoid systems. Wang et al. [98] established an inducible iPSC system co-expressing EGFP and luciferase to track the development of pancreatic islet organoids. This dual-modality strategy fuses cellular-level fluorescence resolution with deep-tissue bioluminescence sensitivity. By integrating the reporters directly into the host genome, this platform establishes an endogenous, self-renewing sensing interface where optical signals scale natively with 3D cellular proliferation and lineage differentiation, completely eliminating exogenous probe depletion, diffusion resistance, or chemical disruption within the culturing matrix. As reviewed by Ovechkina et al. [99], these genetically encoded fluorescent biosensors (GEFBS) allow for the continuous, real-time visualization of signaling pathways and metabolic shifts. The sensing mechanism relies on a chimeric architecture pairing an analyte-specific sensing domain with a non-cytotoxic fluorescent reporter, driving high-dynamic-range signal transduction. By incorporating fluorescent protein genes directly into the host genome, GEFBS eliminate a critical bottleneck in drug screening: false-positive results stemming from overlooked drug bioavailability constraints within specific sub-cellular compartments. The biosensors are synthesized endogenously by the resident cells, thereby functionally replacing the conventional physical sensing substrate with the surrounding extracellular matrix, whose fundamental material properties directly regulate the operation of the sensors without disrupting the native 3D cell-matrix interactions essential for high-fidelity disease modeling.

Spectroscopic and fluorescent techniques offer complementary insights for organoid evaluation, yet present a clear trade-off between label-free global characterization and specific, high-resolution localization. Raman spectroscopy leverages intrinsic molecular “fingerprints” for label-free, long-term tracking of global metabolic trajectories, including proteins and lipids. However, its inherently small scattering cross-section yields weak signals susceptible to matrix interference, and its slow scanning speed precludes the capture of transients. Conversely, fluorescence monitoring, driven by exogenous probes, bioorthogonal chemistry, or genetically encoded reporters, delivers high signal-to-noise ratios and subcellular specificity, making it the premier choice for tracking transient ion fluxes and specific pathway dynamics. Nonetheless, it remains constrained by photobleaching, potential cytotoxicity, and batch-to-batch heterogeneity induced by genetic transduction. Furthermore, the technical limit of optical evaluation lies in the interfacial conflict between device material properties and tissue physical boundaries. First, the high-aspect-ratio structure of 3D organoids induces severe light scattering and attenuation, causing focal plane misalignment and sensitivity degradation in deep tissues. Second, high-resolution, non-destructive imaging relies heavily on rigid windows, such as quartz and PMMA, featuring low autofluorescence and high transmittance. The requirement creates an irreconcilable mechanical mismatch with the ultra-soft, flexible matrices necessary for maintaining organoid morphology or simulating physiological stress. Consequently, future research must focus on hybrid multimodal optical paths and the development of interface materials that balance high transparency and a low refractive index with biomimetic mechanical compliance, thereby maximizing data extraction while preserving physiological fidelity.

### 3.3 Mechanical and physical monitoring

Mechanophysical characterization is essential for evaluating the functional maturity and contractile dynamics of organoids, particularly in cardiac and muscular models. Traditional atomic force microscopy (AFM) remains a gold standard for high-sensitivity force transduction. Svěrák et al. [100] combined AFM with a dual-organoid system to achieve synchronous monitoring of independent cardiac organoids. This setup resolved microscale force variations with nanonewton resolution, capturing vertical contraction amplitudes and lateral mechanical propagation with loads exceeding 30 mN. By detecting delayed contraction peaks and stochastic fluctuations under pentanol treatment, the system effectively distinguished focal rhythm abnormalities from conduction arrhythmias. However, monitoring modalities based on AFM rely on rigid cantilever probes and localized tactile scanning, which exhibit a fundamental incompatibility with suspended 3D organoids. Because these 3D cellular structures are highly compliant, they are exceedingly prone to physical displacement or structural damage under such rigid point-loading. To circumvent these bio-interfacial and alignment limitations, while simultaneously addressing the narrow dynamic range of conventional AFM, Watanabe et al. [101] developed a quartz crystal resonator (QCR)-based probe tailored for automated screening. The underlying technical principle relies on contact resonance mechanics; upon tip-tissue contact, the mechanical elasticity of the 3D tissue shifts the acoustic-mechanical impedance of the system, which is precisely transduced into variations in the resonant frequency (Δf) and quality factor (Q). Benefiting from ultra-low intrinsic frequency noise, this platform expands the dynamic measurement range across five orders of magnitude (nN to 30 mN) with a force resolution of 272 nN. The structural mechanism provides the extreme sensitivity required to probe ultra-soft organoid matrices (2.51 kPa) while preserving the structural rigidity necessary to withstand high loads from stiff or fibrotic tissue domains (1.19 MPa).

The integration of electromechanical coupling provides a more comprehensive readout of organoid physiology by synchronizing contractile forces with bioelectric activity. To investigate drug-induced cardiotoxicity, Yin et al. [102] integrated resistive skin sensors with 3D MEA. The system design features a biting-inspired sandwich configuration where a micromanipulator precisely positions a dynamically beating cardiac organoid between a top resistive skin and a bottom 3D microneedle array, creating a reliable contact “sweet spot”. To optimize this bio-electronic interface, the resistive skin employs an ultra-compliant conductive composite engineered for strict mechanical impedance matching with the ultra-soft neonatal-like cardiac tissue. This ultra-low stiffness allows the sensor to undergo seamless, compliant interfacial deformation under contraction without clamping or inhibiting the organoid's spontaneous, intrinsic beating.

Despite these advances, limitations remain, such as AFM relies on rigid probes unsuitable for 3D suspended organoids; micropillar arrays and micro-indenters have limited dynamic range and slow acquisition; QCR and resistive skin/3D MEA systems often provide only local or planar measurements and require precise contact. Addressing these challenges, Lyu et al. [103] developed a non-invasive nanocracked platinum membrane sensor integrated directly into the culture chamber. The biomimetic design replicates the tactile sensing of a human finger probing an artery pulse, establishing a non-destructive organoid-diaphragm interface through an ‘AFM-like’ soft engaging process. The sensing layer features an ultrasensitive nano-cracked platinum film, where the underlying transduction mechanism relies on the microstructural disconnection-reconnection of platinum nano-islands. When the suspended organoid beats spontaneously, the generated minute fluidic pressure waves or direct vertical displacements are captured by the compliant diaphragm, exponentially amplifying the contact resistance across the nano-cracks to yield an extreme gauge factor >2000.

**Fig. 5:**
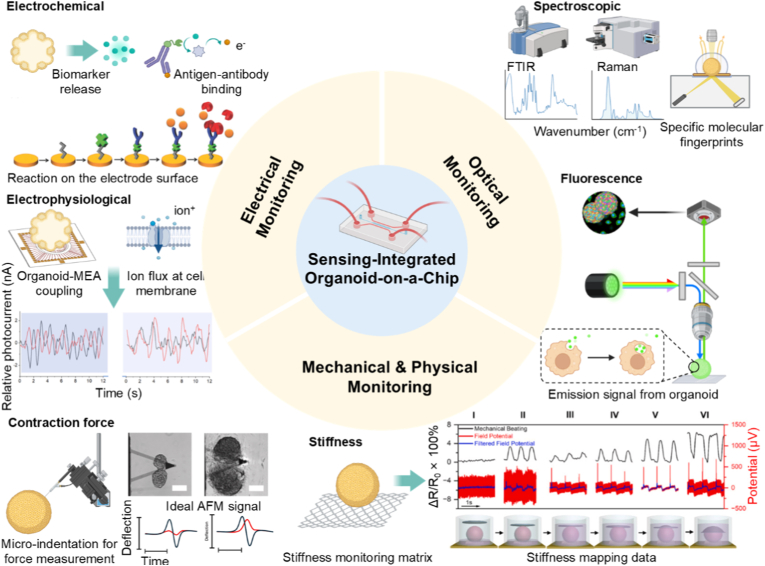
Technical pathways for real-time in situ monitoring in organoid-on-a-chip platforms, including electrical monitoring of electrochemical and electrophysiological signals, optical monitoring via spectral analysis and fluorescence imaging, and mechanical/physical monitoring of contractility, stiffness, and strain.

Although mechanical characterization is indispensable for evaluating the contractile kinetics of cardiac and muscular circuits, intrinsic device intervention universally suffers from fundamental bottlenecks, namely interfacial mechanical damage and 3D anisotropic mapping. During the micromanipulation alignment within probe-integrated micromechanical chips, excessive physical compression, driven by the severe rigid-to-soft Young's modulus mismatch, restricts spontaneous tissue expansion and induces cellular ROS accumulation. To circumvent this, leveraging nanocrack-structured platinum films to capture faint fluidic pressure waves or vertical displacements generated by cell beating establishes an “AFM-like”, ultra-compliant sensing interface. While this modality successfully mitigates direct mechanical damage and modulus-mismatch-induced stress, acquiring complex 3D anisotropic vector data from such an isotropic testing configuration remains a challenge. Nevertheless, the shift demonstrates that future mechanical chip integration is rapidly moving away from rigid, invasive probes toward fully 3D-enveloping, low-toxicity, and bio-adaptive ultra-soft flexible electronics. By bridging the gap between mechanical force and bioelectric activity, these integrated sensing strategies establish a high-fidelity foundation for real-time functional evaluation and drug response monitoring on-chip (Fig. 5), with their specific performance benchmarks and technical modalities summarized in Table 3.

**Table 3:** Performance parameters and technical modalities for in situ monitoring of organoid functional dynamics.

Modality	Analyte	Technology	Performance parameters (LOD/Sensitivity/Range)	Long-term stability	Sensing interface and depth	Throughput and scalability	Ref
Electrochemistry	ALB	Microfluidic electrochemical	LOD: 0.09 ng/mL; Sens: 1.35 (log (ng/mL))^−1^; Range: Logarithmic scale response	Days	Superficial/Non-invasive	High	[76]
Electrochemistry	5-HT	3D-printed carbon/PLA	LOD: 0.07 μM; Sens: 13.1 nA/μM; Range: 0.1–2 μM	Days	Superficial/Non-invasive	High	[77]
Electrochemistry	Kidney cells	Conductive surface signature	LOD: 21363 cells; Signal: ∼10^−4^ μA/cell; Range: 2.14 × 10^4^–1.00 × 10^6^ cells	Days	Superficial/Non-invasive	Medium	[78]
Electrochemistry	Glutamate	Enzyme-modified microelectrode	LOD: 5.6 μM; Sens: 93 nA μM^−1^ cm^−2^; Range: 5 μM–0.5 mM	Hours	Superficial/Non-invasive	High	[80]
Electrochemistry	α-synuclein	Molecularly imprinted polymer	LOD: 10 fg/mL; Sens: 9 fg/mL; Range: 1.57–63.8 pg/mL	Hours	Superficial/Non-invasive	Medium	[81]
Electrophysiology	Cardiac APs	Shape-adaptive shell MEA	Slope-based accuracy: 0.875	Weeks	Interfacial/Semi-invasive	Medium	[86]
Electrophysiology	Neuronal networks	Magnetically reconfigurable MEA	Resolution: 0.002°; Detectable area increase: 891×	Weeks	Interfacial/Semi-invasive	Medium	[88]
Spectrum	Biochemical markers	Confocal raman spectroscopy	Amide I (∼1650 cm^−1^), DNA (∼785 cm^−1^)	Hours	Deep-tissue/Non-invasive	Low	[91]
Fluorescence	Lysosomal pH	Lyso-BFP	Excitation: 405–450 nm; Emission: 460–500 nm	Hours	Deep-tissue/Non-invasive	High	[96]
Mechanophysica	Contraction force	AFM transducer	Resolution: Nanonewton; Max Load: >30 mN	Hours	Deep-tissue/Invasive	Low	[100]
Mechanophysica	Stiffness/Force	QCR-based probe	Resolution: 272 nN; Range: 5 orders of magnitude	Days	Superficial/Non-invasive	Low	[101]
Mechanophysica	3D beating force	Nanocracked Pt membrane	Gauge Factor >2000; Nanonewton sensitivity	Days	Superficial/Non-invasive	Medium	[103]

### 3.4 Organ-specific sensing requirements and tailored platform designs

Sensing-integrated organoid-on-a-chip platforms cannot rely on a single, generalized electrode architecture for blind monitoring. Instead, the distinct biological characteristics of organ models strictly dictate the customized design of sensing interface materials, device physical structures, and temporal resolutions. First, for parenchymal or epithelial organoids characterized by high metabolic flux or specialized secretory functions, such as liver, kidney, lung, and intestine, the primary objective of real-time sensing is the non-destructive tracking of spontaneous, continuous biochemical fluxes. For instance, hepatic organoids continuously synthesize functional proteins (ALB, α1-AT) [104,105] and generate metabolic wastes (urea, bile acids) [106,107]. Renal organoids inherently express functional proteins (megalin) [108] and specific transporters (OAT1, OCT2) [109] to maintain reabsorption. Lung organoids specifically secrete surfactant proteins (SP-B, SP-C) [110,111] and high-molecular-weight glycoproteins (MUC5AC) [112] for surface tension regulation, while intestinal organoids autonomously synthesize apolipoproteins (ApoB-48) [113], barrier-maintaining mucus (MUC2) [114], and neurotransmitters (5-HT) [115]. To capture these unique chemical signatures, the sensor interfaces must be deeply customized. High selectivity can be achieved by functionalizing electrodes with antibodies or aptamers, or by designing electrode materials with targeted redox catalytic capabilities. Moreover, charge accumulation effects can be pioneered via FETs paired with selective porous membranes, or through target-specific fluorescent nanoparticles for non-invasive optical tracking. Simultaneously, to combat severe biofouling and baseline drift caused by high-serum, protein-rich culture media over multi-week cultivation cycles, anti-fouling strategies, such as the incorporation of zwitterionic coatings or the integration of automated microfluidic pneumatic valves for in situ dynamic washing, must be deployed to maintain interfacial sensitivity.

Second, during barrier epithelium maturation or musculoskeletal tissue development, platforms must adapt to a drastic evolution in intrinsic physical properties. In barrier-forming models, such as intestinal and blood-brain barrier organoids, cellular polarization and tight junction assembly manifest electrically as a continuous increase in TEER [116,117]. However, during this intense structural remodeling, the substantial traction forces generated by cells often induce severe volume contraction and deformation. This dynamic physical shifting frequently causes the tissue to delaminate from fixed underlying microelectrodes or drift out of the effective sensing plane, leading to severe motion artifacts or complete signal loss. Consequently, impedance and mechanical sensing platforms require compliance-matched engineering. This is achieved by tuning the initial crosslinking density of the culture matrix to smoothly dissipate local contractile stresses, fabricating electrodes from flexible materials with high mechanical compatibility, and employing microstructural boundaries, such as microgrooves or elastic shells, to confine the contracting tissue within the electromagnetic sensitive field or the precise optical focal plane, thereby eliminating measurement distortion from geometric drifting.

Finally, for excitable tissues such as cardiac, neural, and muscular organoids, functional phenotypes are defined by millisecond-scale electrophysiological transients, such as action potentials or local field potentials, and spontaneous mechanical beating [[118], [119], [120]]. Capturing these high-frequency dynamics requires a high-fidelity bio-interface. Traditional rigid electrode arrays, including silicon, gold, or platinum, exhibit an extreme young's modulus mismatch against soft organoid matrices. This mismatch inflicts irreversible mechanical tearing, restricts spontaneous tissue contraction, and induces cellular ROS accumulation, which distorts physiological fidelity and accelerates apoptosis. To circumvent these limitations, sensing architectures must move away from rigid boundaries toward space-adaptive flexible electronics. Utilizing open-mesh flexible microfilament structures or 3D-printed liquid metals allows the sensing interface to achieve dynamic, conformal co-localization with the internal microdomains of the organoids. Combined with low-impedance, biocompatible surface modifications, these systems capture multi-channel, high-frequency, and high-signal-to-noise ratio signals while fully preserving the structural and biological integrity of the living tissue. The correlations among model-specific pathophysiological features, targeted sensing modalities, and custom-engineered platform parameters are comprehensively compiled and contrasted in Table 4. Concurrently, to offer a consolidated pipeline mitigating the baseline drift and bio-macromolecular fouling detailed across these models, the relative merits, intrinsic limitations, and demonstrated efficacy of mainstream anti-biofouling coatings are systematically compared in Table 5.

**Table 4:** Organ-specific sensing requirements and tailored platform designs.

Organ system model	Distinct physiological characteristics	Sensing target and modality	Tailored sensor material and interface engineering	Device structure	Temporal resolution
Hepatic and renal	High metabolic flux; Endogenous metabolite synthesis; Specific transporter dynamics	Biomarkers; Transporter activity; Continuous biochemical flux	Aptamer/antibodyfunctionalized electrode; Zwitterionic anti-fouling coatings; Redox-catalytic nanomaterials	Integrated microfluidic networks with pneumatic valves	Minutes to hours
Pulmonary and gastrointestinal	Barrier epithelial secretion; Macromolecular glycoprotein flux; Surfactant/neurotransmitter release	Mucins; Surfactant proteins; Dynamic barrier permeability	Selective porous membranes; Charge-sensitive FETs; Target-specific fluorescent nanoparticles	Non-invasive optical/bioelectrochemical interfaces	Minutes to continuous
Barrier and musculoskeletal	Dynamic tissue remodeling; Matrix deposition; Drastic Young's modulus shift	TEER; Interfacial traction force; Structural contraction	High-compliance flexible substrates; Stretchable conductive polymers; Tunable hydrogel crosslinking density	Micro-grooved arrays and elastic encapsulations; Adaptive geometric boundaries	Hours to days
Cardiac, neural and muscular	Millisecond-scale bioelectric transients; Spontaneous, high-frequency mechanical beating	Action potentials; LFP; Contractile kinetics	Low-impedance biocompatible coatings; Liquid metals; Ultra-soft conductive hydrogels	3D open-mesh flexible microfilament arrays; Conformal 3D-enveloping interfaces	Millisecond

**Table 5:** Comparison of interfacial anti-biofouling strategies for biosensing platforms.

Strategy type	Anti-fouling modality	Primary merits	Intrinsic limitations	Anti-fouling performance	Ref
Active cleaning	Active magnetic washing	Provides precise, on-demand surface washing; zero chemical toxicity; extends operational lifespan	Requires peripheral magnetic fields and complex architecture; induces transient signal disruption during washing; risks disturbing localized microenvironments	Achieves ∼100% on-demand recovery of active area by mechanically dislodging chemisorbed proteins; maintains long-term interfacial sensitivity	[121]
	Automated fluidic flushing	Enables passive, capillary-driven chronological rinsing; eliminates risks of chemical or biological contamination to tissues	Risks damage to delicate analytes under shear; requires complex microfluidic architecture; induces transient signal disruption during washing; ineffective against rigidly chemisorbed proteins	Suppresses initial non-specific binding by maintaining continuous fluidic shear stress during passive sample transport	[122]
Physical structural design	Superhydrophobic structures	Minimizes real contact area via the plastron effect; exhibits exceptional water contact angles and rolling adhesion to prevent initial attachment	Dramatically reduces effective contact sensing area, compromising sensing sensitivity and accuracy; trapped air layers easily collapse under continuous microfluidic pressure or surfactant-rich culture media	Achieves remarkable self-cleaning and corrosion resistance by micro-topological nanoparticle networks; highly robust against severe mechanical damage	[123]
	Sacrificial layers	Periodically sheds the outermost fouled strata to continuously renew the pristine sensing interface	Mandates high-frequency recalibration of standard curves due to variations in shifting interfacial thickness; induces continuous monitoring data gaps during the erosion/re-assembly cycles	Enables biosensors to safely operate over dozens of cycles in severely fouling fluidic streams under an automated, batch-style intermittent tracking mode	[124]
Chemical surface modification	Fluorinated coatings	Ultra-low surface energy and exceptional chemical inertness; repels a wide spectrum of sticky biomacromolecules	Suffers from poor adhesion to substrates, requiring harsh chemical/thermal curing; forms dense passive barriers that slow down sensing kinetics	Maintains stable continuous-flow biosensing with negligible drift even when exposed to undiluted, heavily fouling matrix blood samples; ensures high baseline fidelity across multi-day assays	[125]
	Zwitterionic coatings	Forms a dense, electrostatically bound hydration barrier; exhibits superior long-term stability against chemical degradation under prolonged fluidic flow	Requires complex, multi-step surface polymerization; severe hydro-swelling risks encroaching microfluidic channels and physical screening of sensitive footprints, which impairs fluidic control and signal transduction	Achieves ultra-low protein adsorption with near-zero baseline drift in undiluted plasma; maintains long-term signal fidelity across multi-week tracking	[126,127]
	PEGylation	Establishes a flexible, well-defined hydration barrier that sterically repels non-specific bio-macromolecules; highly cost-effective and compatible with nano-structured sensitive substrates	Highly susceptible to oxidative degradation in complex physiological fluids under long-term fluidic shear, leading to irreversible chain cleavage and baseline drift over extended periods	Enables ultra-sensitive bio-sensing with near-zero interference within raw human serum by shielding active spaces; best suited for acute or intermediate tracking windows	[128,129]
	Natural macromolecule passivation	Facile, equipment-free “one-drop” coating; leverages unique antibody-protein synergy to maximize repellent sites	Passive desorption over time; requires routine re-blocking; potential cross-reactivity with complex biosensors	Reduces non-specific adsorption to near-zero; effectively eliminates matrix background noise across high-sensitivity assays	[130,131]

## 4 Real-time sensing-integrated organoid-on-a-chip: multidimensional applications

The integration of diverse sensing modalities into organoid-on-a-chip platforms provides a systematic approach for characterizing complex biological systems in vitro. By enabling the continuous acquisition of physiological data, these integrated platforms facilitate a more detailed understanding of tissue dynamics compared to traditional static models. This section reviews the multidimensional applications of these systems, beginning with the reconstruction of fundamental physiological and metabolic processes. We then discuss their utility in elucidating pathological mechanisms and accelerating drug screening and personalized therapeutics. Finally, the application of sensing-integrated interfaces in monitoring neural network activity and brain-inspired computing is highlighted. These applications demonstrate the potential of sensing-integrated organoid-chips to enhance the predictive power of in vitro models for clinical and industrial use.

### 4.1 Reconstruction of physiological and metabolic processes

**Fig. 6:**
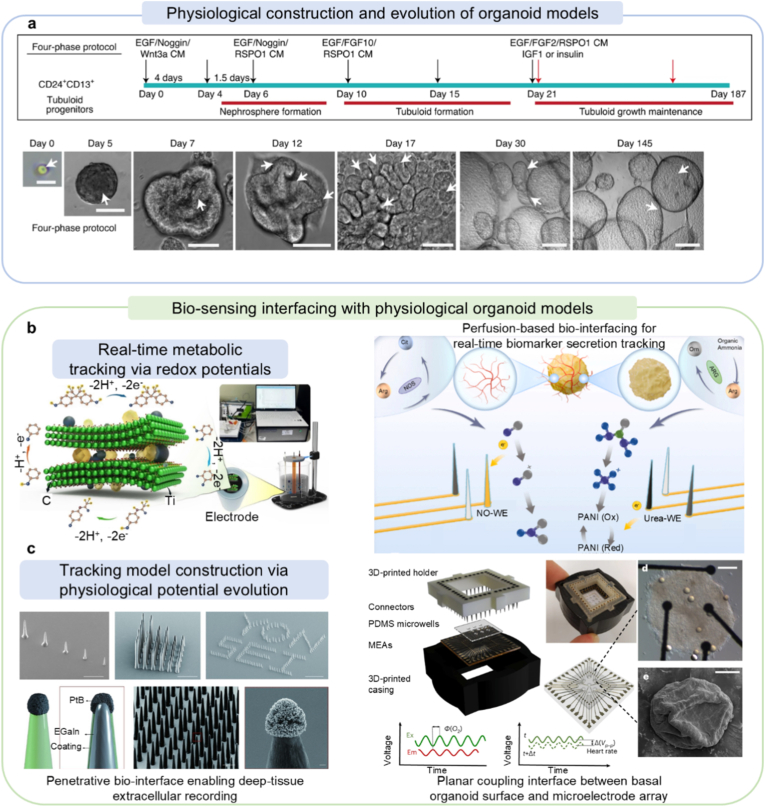
Construction of physiological models and biosensing integration in organoid-on-a-chip systems. (a) Workflows for constructing renal and hepatic physiological models, illustrating the compartmentalized reconstruction of renal and hepatic physiological models [132], copyright 2022, Springer Nature, license: 6285781424112; (b) Real-time tracking of organoid development using embedded electrochemical sensors, showing the real-time tracking of organoid developmental maturation via embedded electrode arrays [133,134], copyright 2026, 2025, Elsevier, license: 6285790462718, 6285791128147, and (c) 3D bio-electrophysiological platforms, demonstrating the spatial wrapping of organoids using flexible 3D microelectrode networks for multi-site neural/cardiac signal capturing [135,136], copyright 2023, Springer Nature, license: 6285800819607; copyright 2025, American Chemical Society, license: 6285810367578.

Establishing a quantitative baseline of how organoids recapitulate physiological processes is a strict prerequisite before implementing real-time monitoring, as this dynamic biological knowledge directly instructs the rational material selection and interfacial parameters of sensing platforms (Fig. 6a). In cardiac research, Kim H. et al. [137] reconstructed key stages of human cardiogenesis using multicellular hPSC-derived models to track cardiomyocyte (cTnT, α-actinin), endothelial (CD31/PECAM-1) and fibroblast (vimentin/FSP1) markers alongside electrophysiological signatures like rhythmic beating. Similarly, in intestinal organoid systems, Gjorevski et al. [138] demonstrated that initial epithelial geometry predictably governs crypt-villus patterning and regionalized differentiation, as evidenced by the spatially resolved expression of Lgr5-GFP, Lysozyme, and CK20/MUC2. Expanding beyond these primary models, other robust organ systems have successfully established structural and secretory baselines, including the pancreas, biliary epithelium, and kidney. For instance, the hPSC-derived pancreatic organoids validating exocrine/ductal maturation via post-transplantation enzyme activity [139], cholangiocyte organoids reconstructing transport architectures within biomaterial scaffolds [140]. Under fluid shear stress within a microfluidic platform, blood vessel organoids self-assembled into perfusable microvascular networks [38]. The tubular polarity and structural integration were successfully confirmed via PECAM1, MCAM, and PODXL markers.

To overcome the limitations of isolated tissue modeling, advanced multi-organoid platforms have expanded into system-level endocrine interactions. For instance, Sun et al. [141] developed a microfluidic gut-islet axis chip using biomimetic hydrogel scaffolds. Under continuous perfusion, the platform replicated the glucose-regulatory feedback loop, tracking real-time GLP-1 and insulin signaling during glucose fluctuations. Similarly, Tao et al. [142] extended this strategy to a circulatory liver-islet axis chip. By co-culturing hiPSC-derived liver and islet organoids for 30 days, the platform successfully captured the bidirectional feedback loop where glucose-stimulated insulin secretion drives downstream hepatic glucose utilization and glycogen synthesis.

Beyond soft tissues, bone and joint organoids offer critical platforms for mechanobiological and regenerative studies. Hu et al. [143] highlighted how these models recapitulate matrix formation across the vascular-cartilage-bone unit by monitoring lineage-specific markers including COL2A1, SOX9, and ALP. To guide this structural maturation, advanced bio-fabrication strategies have emerged. Chen et al. [144] employed a DNA-reversible cross-linking strategy to 3D-print viscoelastic cartilage organoids, while Li et al. [145] utilized enzyme-catalyzed mineralization to reconstruct the temporal sequence of endochondral ossification, tracked via RUNX2 and OCN transitions.

Parallelly, neural organoids provide foundational blueprints for neuro-simulation and brain-inspired computing. Giandomenico et al. [146], Birtele et al. [27], and Amin N. et al. [147] demonstrated that tracking dynamic cellular shifts can map human corticogenesis from pluripotent neuroepithelium to layered neuron-glia networks, sequentially validated via progenitor (PAX6, TBR2) and mature cortical (CTIP2, SATB2, and S100β) markers. Expanding into sensory signaling, Hu et al. [68] established cochlear organoids expressing hair-cell markers Myo7a and phalloidin, acquiring inner-ear mechanoelectrical properties under physical stimulation. Similarly, Bannier-Hélaouët et al. [148] developed the human conjunctiva organoid model at an air-liquid interface by tracing NGFR^+^ basal progenitors and MUC5AC^+^ goblet cells, capturing secretory responses under immune cues like IL-4/IL-13. Crucially, despite past advances in organoid models and on-chip engineering, the tracking of these diverse lineage-specific markers and dynamic cellular shifts remains heavily reliant on conventional endpoint assays, which obscure the continuous biophysical transitions and rapid functional fluctuations that define organ development.

To resolve this temporal blindness and eliminate the sample-to-sample variations inherent to destructive tissue extraction, the strategy must shift toward continuous in situ characterization. Integration of metabolic biosensing into organoid-on-chip systems further enables dynamic, quantitative tracking of organ development and function. For optimizing transplantation readiness in hepatic organoids, Sun et al. [133] developed a sensor-integrated organoid-chip that simultaneously monitored vascularization and liver metabolic performance, providing real-time readouts of organoid maturation (Fig. 6b). The embedded poly (eugenol)-based microneedle electrochemical sensor within the chip monitored nitric oxide, capturing the endogenous NO gas molecules released by vascular endothelial cells during their self-organized networking process, while an integrated enzyme-catalyzed electrochemical sensor modified with polyaniline converted the fluctuations in local ionic concentration and charge density generated by urea hydrolysis into electrical signal outputs. The temporal profiles revealed a critical inflection point on day 5 marking the convergence of vascular integration and metabolic competence, defining the optimal transplantation window subsequently validated in a mouse liver-cirrhosis model. By utilizing this integrated sensing technology, they broke through the limitations of conventional “black-box” organoid culture, clearly elucidating and quantifying how the microfluidic environment regulates multicellular self-organized development, and discovering the convergence inflection point of biochemical signals to provide crucial decision-making guidance for clinical transplantation. Crucially, from a clinical diagnostic perspective, the developmental inflection points calibrated by this sensing platform perfectly map onto the clinical gold standards used to evaluate graft maturity, including the determination of synthetic baselines via total serum protein/ALB assays, profiling detoxification systems using indocyanine green clearance or dynamic blood ammonia measurements, and quantifying microvascular perfusion through Doppler ultrasound. The in situ captured urea flux and NO release by the sensors point-to-point align with these clinical metabolic detoxification and vascular perfusion functions at the underlying physiological level. Consequently, this platform provides a non-destructive, quantitative, and intrinsic developmental surrogate biomarker well before macroscopic imaging alterations manifest.

Moving beyond macromolecular chemical metabolic fluctuations toward high-frequency electro-mechanical activities, integrated microphysiological systems must fundamentally resolve the multiphysics coupling at the tissue-sensor boundary. In cardiac organoids, M. Ghosheh et al. [135] precisely tuned matrix stiffness and anisotropic stress to guide the self-organization of human cardiac tissue within a 3D scaffold, generating multi-chambered, vascularized cardiac organoids that not only replicate chamber-level morphology but also exhibit synchronized electrophysiology, mechanical contraction, and metabolism-coupled function. From an interfacial engineering standpoint, tuning the scaffold matrix's Young's modulus to establish physiological anisotropic stress not only guides the highly synchronized biological maturation of cardiac networks but also directly dictates the signal-to-noise ratio of integrated transducers. By utilizing tissue-conformable micro-flexible electrodes, flexible strain sensors, and solid-state micro-electrochemical sensors integrated at the interface between the microfluidic channels and the tissue, they achieved in situ, simultaneous recording of oxygen consumption rate, extracellular field potential, and contractile strain at a frequency higher than 10 Hz, thereby attaining sub-second temporal resolution. Integrating tissue-conformable micro-flexible electronics and strain sensors to match the soft, dynamic compliance of the contracting cardiac tissue fundamentally circumvents the rigid-to-soft mechanical boundary mismatch, effectively absorbing continuous kinetic shear strain to eliminate motion-induced capacitance fluctuations and measurement artifacts. These measurements uncovered a 1 Hz metabolic respiratory cycle synchronized with the organoid's rhythmic electrical activity, driven by mitochondrial Ca^2+^ oscillations rather than mechanical contraction, establishing an integrated multimodal sensing framework for functional assessment of vascularized cardiac organoids. The flexibility and low-impedance design of the materials guaranteed the physical signal quality of the sensing interface, whereas the modulation of anisotropic stress via microenvironmental engineering ensured the macroscopic synchronization of the biological responses. Complementarily, Park et al. [136] developed a soft 3D bioelectrode platform using liquid-metal printing to resolve the critical material design logic and mechanical mismatch at the sensing-matrix interface. Unlike conventional rigid conductors that generate severe interfacial shear stress and cellular apoptosis on soft tissues, this approach leveraged gallium-based liquid metal to fabricate geometrically customizable, tissue-compatible electrodes capable of dynamically conforming to the organoid's 3D internal regions and contractile motion. To circumvent the sensing bottleneck of high interfacial impedance, platinum nanostructures were integrated at the electrode tips to enhance electrochemical transduction without sacrificing flexibility. From an interfacial materials perspective, matching the tissue-comparable compliance of organoids fundamentally suppresses localized mechanical shear strain and cellular detachment during rhythmic beating, thereby safeguarding baseline signal stability over an extended 21-day cultivation period. Concurrently, insulating the electrode sidewalls with a parylene-C encapsulation layer restricts the active electrophysiological window, while the electroplated platinum nanostructures drastically expand the electrochemical surface area to minimize charge-transfer resistance at the bio-interface. The configuration, coupled with the high intrinsic conductivity and fluidic deformability of the liquid metal core, effectively dampens motion-induced capacitance fluctuations and electronic background noise in dynamic aqueous environments. This design enabled high-throughput parallel electrocardiography (ECG) recording across 32 organoids, capturing electrical signatures during development as well as drug-induced alterations in cardiac rhythm and action-potential dynamics with a 3-fold signal-to-noise ratio improvement. From a clinical perspective, the cardiac rhythm and contractile strain captured by these two chips deeply align with the clinical gold standards of ECG and echocardiography used to evaluate cardiac development. At the underlying cellular level, they respectively map onto core functional indicators in clinical diagnosis such as electrophysiological conduction including arrhythmic evolution and myocardial mechanical pumping exemplified by the shortening fraction (Fig. 6c).

For brain organoids modeling regional interactions, Ozgun et al. [149] cultured midbrain and striatal organoids via surface-growth methods and induced them to establish a self-assembled dopaminergic inter-organoid pathway, forming a functional midbrain-striatal connectivity model. Incorporation of a MEA allowed real-time monitoring of synaptic firing, spike synchrony, and inter-organoid signal propagation, while calcium imaging revealed coordinated activity between dopaminergic and GABAergic neuronal populations, confirming functional coupling between the two regions. Upon exposure to the dopaminergic neurotoxin 6-OHDA, the connected pathway model exhibited heightened electrophysiological sensitivity compared to isolated midbrain organoids, demonstrating the platform's capacity to emulate pathological perturbations and quantify network-level dysfunction, thus enabling experimentally tractable investigation of complex neural circuitry and disease-relevant signaling pathways in vitro. From a clinical perspective, the real-time tracking of synchronized firing across distinct brain regions closely corresponds to the clinical gold standard of using functional magnetic resonance imaging (fMRI) or diffusion tensor imaging to evaluate brain functional connectivity. The synchronized bursting and signal propagation velocity captured by MEAs directly map onto the underlying electrophysiological essence of functional pathway maturation within brain networks at the microscopic scale.

### 4.2 Disease modeling and pathological mechanisms

**Fig. 7:**
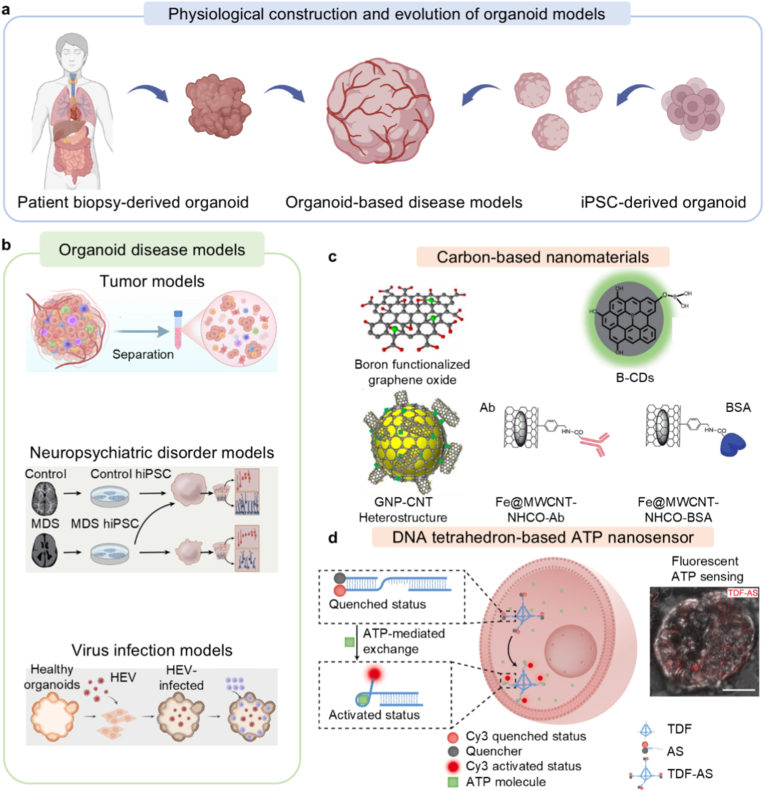
Integrated sensing for disease modeling and pathological mechanism studies in organoid-on-a-chip. (a) Schematic for isolating patient biopsy-derived or iPSC-derived organoids establish disease models; (b) Biomedical classification of major organoid pathological systems, demonstrating representative platforms for common tumor, neuropsychiatric, and viral infection models [147,150,151], copyright 2018, Elsevier, license: 6285831239019; published under CC BY license; copyright 2025, Springer Nature, license: 6285830955362; (c) Application of nano-architectures for carbon-based sensors, showing the structural configuration of boron-functionalized graphene oxide, carbon dots, and multi-walled carbon nanotube heterostructures for antibody/BSA immobilization [22], copyright 2025, Elsevier, license: 6285840239056; (d) Intracellular signaling pathways of DNA tetrahedron-based ATP nanoprobes, illustrating the ATP-mediated molecular replacement mechanism from a quenched status to an activated fluorescent state during dynamic pathological monitoring [152], copyright 2023, Elsevier, license: 6285840483303.

In disease modeling, organoid technology serves as a high-fidelity in vitro platform for reconstructing tissue-specific architectures and functional states [16] (Fig. 7a). Currently, disease modeling predominantly relies on two complementary cell-based systems, each presenting distinct trade-off. On one hand, primary tissue-derived systems, such as patient-derived adult stem cells or tumor-derived primary organoids (PDOs), faithfully preserve the native clinicopathological heterogeneity and microenvironmental niches of individual patients [153,154]. However, their limited long-term expansion capacity in vitro constrains high-throughput scalable applications. On the other hand, renewable systems possessing infinite replicative capacity, such as hPSCs and iPSCs, offer robust multi-lineage differentiation potential. The renewable models are highly compatible with genome-editing tools like CRISPR/Cas, enabling the precise introduction of defined pathogenic mutations to reconstruct personalized genetic backgrounds and model evolving pathological initiation from scratch [155].

In tumor modeling, PDOs function as sophisticated “patient avatars” by integrating immune cells, stromal components, and biochemical cues into complex tumor niches, thereby facilitating the study of drug resistance, precision oncology, and high-throughput screening [156,157]. For instance, Lv et al. [150] demonstrated that PDOs retain key molecular hallmarks and driver mutations of parent tumors, such as KRAS, TP53, BRAF, effectively reproducing tumor initiation and therapeutic response. To standardize these models for scalable applications, Wang et al. [158] and Xu et al. [159] leveraged acoustofluidic platforms for tunable microdroplet assembly to generate uniform tumor organoids. By monitoring surface markers (EpCAM, Ki-67, CD44/CD133) and cleaved-caspase-3, they transformed static observations into a quantifiable evolution of tumor self-organization and apoptosis. Furthermore, this predictive capacity extends to evaluating drug-induced liver injury (DILI) [160]. By profiling functional metabolic markers (ALB, CYP3A4) against hepatic injury indicators (ALT, AST) and cell-death traces (TUNEL, cleaved-caspase-3), the system quantitatively captured the fluctuating trajectories of drug exposure and injury-to-recovery dynamics. As illustrated in Fig. 7b, tumor tissues can be separated and cultured into organoid models for drug responsiveness assessment.

In chronic disease and natural aging modeling, organ stiffening and excessive scarring are critical biophysical hallmarks arising from abnormal extracellular matrix accumulation. Organoid models have successfully reconstructed these mechanical shifts across various tissue types. For instance, Sun et al. [161] utilized cartilage organoids to reveal that osteoarthritic inflammation triggers miR-24 downregulation and TAOK1 activation, leading to cell ferroptosis and cartilage degeneration. Similarly, multi-organ platforms [162] identified hepatocyte-derived 27-hydroxycholesterol as a circulating driver that induces bone senescence and bone loss. In biliary and renal systems, Soroka et al. [163] and Gupta et al. [164] demonstrated that persistent immune-inflammation and circulating factors induce pro-inflammatory phenotypes and abnormal matrix deposition, recapitulating glomerular and biliary sclerosis. Genetic and viral factors also contribute to these structural shifts. Guan et al. [165] revealed that PKHD1 mutations trigger a TGF-β mediated transition in cholangiocytes, leading to the formation of thick collagen fibers and a significant rise in matrix modulus. Concurrently, Xu et al. [132] leveraged CRISPR-engineered PKD1/PKD2 knockouts in adult-derived kidney tubuloids to model ADPKD cystogenesis. Beyond genetic determinants, Jansen et al. [166] proved that SARS-CoV-2 directly infects kidney cells and initiates pathological repair programs, driving the explosive expression of Collagen Ⅰ and the transition from acute inflammation to chronic tissue stiffening.

Beyond structural aging, organoid technologies offer sophisticated frameworks for modeling complex neurological [155], inflammatory, and infectious regimes. For neuro-oncology safety diagnostics, Xue et al. [167] established a glioblastoma-like microenvironment within brain organoids to quantify the proliferative capacity and invasive potential of cell products, by monitoring pluripotency markers (Nestin, OCT4) alongside single-cell RNA sequencing (Fig. 7b). Transitioning to mucosal pathologies, intestinal organoids successfully model IBD progression. Quantifying inflammatory cytokines (IL-6, TNF-α and IL-1β) alongside tight-junction markers (ZO-1, Occludin) tracks acute barrier breaches and transient permeability alterations [168]. Parallelly, to capture systemic infectious process, Liu et al. [151] developed macrophage-enhanced liver organoids (MaugOs) to simulate viral entry and immune reaction across HEV, SARS-CoV-2, and MPXV infections, where cell-type-specific expressions (CD68, EpCAM) and viral proteins (ORF2) enabled the precise quantification of infection severity and tissue injury (Fig. 7b).

Despite profound advancements in structural and biological recapitulation, the vast majority of current disease-modeling studies still fundamentally rely on static, endpoint destructive assays. This implementation bottleneck stems directly from specific limitations inherent to particular pathological environments. First, complex structural diseases, such as tissue stiffening, fibro-scarring, and regionalized tumor invasion, are characterized by high spatial heterogeneity. Current commercial sensing modalities predominantly yield only spatial averages, preventing researchers from capturing and resolving early biomarkers or directional mechanical strains prior to global histopathological transitions. Second, in workflows involving infectious diseases and microbial-induced organoid pathologies, stringent biosafety protocols mandate absolute isolation within negative-pressure biosafety cabinets; However, existing sensing configurations are severely constrained by bulky external data acquisition hardware, intricate metal cabling networks, and non-sterilizable components, which poses serious risks of breaching pathogen containment barriers and subsequently forces a reliance on standalone endpoint quantification. Third, acute pathological transitions release an uncontrolled flood of viscous biological matrices, including genomic fragments, viral capsids, cellular debris, bacterial spores, and adhesive inflammatory cytokines, which inevitably induce severe interfacial biofouling and surface passivation on integrated electrochemical and optical transducers, rapidly resulting in baseline drift and sensor failure. Overcoming these critical vulnerabilities represents the next essential frontier within the field of sensing-integrated organoid-on-a-chip engineering.

In the pursuit of highly physiologically relevant disease models, conventional 3D organoids have demonstrated remarkable success in recapitulating tissue architecture and cellular functions. However, a critical bottleneck remains: the lack of non-invasive, real-time methodologies to monitor the internal biochemical and biophysical shifts. This “black-box” limitation hampers the ability to correlate transient microenvironmental fluctuations with long-term pathological phenotypes, often obscuring the early mechanistic drivers of disease. To bridge this gap, transforming static organoid cultures into dynamic, sensing-integrated platforms has become essential for unlocking the full potential of these models in mechanistic studies and drug discovery. Recent work [22] demonstrated that integrating carbon-based nanomaterial sensors, such as graphene, carbon nanotubes, and graphene oxide, into organoid-on-chip systems enables continuous monitoring of cellular metabolism, electrochemical status, and biomechanical responses, providing high-temporal-resolution data for functional validation, mechanistic studies, and drug target discovery (Fig. 7c). Building on this approach, embedding electrochemical or mechanosensitive nano-sensors within organoid chips transforms static observation into dynamic sensing, allowing real-time tracking of metabolic perturbations, ionic flux, mechanical deformation, and cell-matrix force coupling. In tumor and organ metabolic disease models, such integrated sensing platforms reveal the intrinsic relationships between microenvironmental changes, cellular responses, and pathological phenotypes, while also providing real-time feedback and high-throughput potential for drug screening. Precision oncology has further highlighted the value of patient-derived tumor organoids for evaluating multiple potential therapies in vitro prior to systemic treatment. By using freshly obtained tumor biopsies, quantifiable disease readouts, and genomic consistency verification, organoid-based models can correlate in vitro drug responses with clinical imaging and pathology outcomes, such as response evaluation criteria in solid tumors (RECIST) or pCR [169], providing a foundation for combining organoid drug screening with real-time biosensing to improve the clinical relevance of therapeutic evaluation. Traditional tumor assays, such as ATP measurements, rely on cell lysis kits and provide only single-time-point information, limiting continuous monitoring. Zhang et al. [152] addressed this limitation by developing a DNA tetrahedron-based ATP nano-sensor. Since fragile cancer organoids are exceptionally sensitive to foreign chemical agents, employing native DNA as the primary structural material ensures negligible cytotoxicity, and DNA self-assembly is utilized to construct a tetrahedral DNA framework with 3D spatial rigidity. This geometrically rigid topology affords robust steric hindrance that effectively safeguards the nanostructure against intracellular enzymatic degradation; simultaneously, this unique nanoarchitecture significantly enhances cellular membrane permeability, enabling non-invasive and spontaneous penetration into deep-tissue cells for in situ, intracellular ATP detection, enabling up to 26 days of dynamic monitoring in human lung cancer organoids. By seamlessly tracking in situ growth trajectories, the sensor transforms static endpoint data into time-resolved profiles, capturing transient metabolic fluctuations to reveal real-time disease progression (Fig. 7d). Similarly, Ruan et al. [170] integrated vascularized tumor organoid chips with real-time ELISA modules to monitor secreted biomolecules under drug treatment, such as VEGF and IL-6, demonstrating high-fidelity simulation of tumor microenvironments and enabling real-time evaluation of cisplatin and bevacizumab efficacy, advancing both drug screening and personalized therapy strategies. From a clinical perspective, the in situ captured intracellular ATP microscopic metabolic transients and dynamic fluctuations of vascular VEGF and IL-6 secretion by this platform closely correspond to the RECIST for assessing tumor remission and the pCR gold standard. These real-time on-chip data deliver a sensitive response at the earliest onset of cellular functional abnormalities, demonstrating a high correlation with subsequent clinical imaging and pathological outcomes.

Mechanical stimulation under physiologically relevant conditions has also been employed to recapitulate the pathophysiology of genetic diseases. Hiratsuka et al. [171] generated an autosomal recessive polycystic kidney disease organoid-on-chip model by placing PKHD1-mutant renal organoids in microfluidic channels, simulating distal nephron fluid flow and shear stress. This “physical-biological signal coupling” strategy activated mechanotransduction pathways, inducing nephron expansion and upregulation of mechanosensitive molecules such as RAC1 and FOS. By coupling long-term, non-invasive live-cell time-lapse imaging for macroscopic cyst tracking with multi-time-point z-stack confocal profiling, this platform achieved high-content, segment-specific 3D quantitative mapping of dynamic tubule dilation trajectories to decipher complex disease pathologies and monitor progression. Conventionally, diagnosing and tracking autosomal recessive polycystic kidney disease rely on abdominal ultrasound or MRI to monitor total kidney volume alongside serum creatinine to estimate the glomerular filtration rate. Intriguingly, this organoid platform captures early luminal expansion mechanical signals well before such macroscopic tubule cysts emerge to determine pathological progression. The microscopic morphological lesion directly reflects the aberrant activation of RAC1 and FOS molecular pathways, which subsequently drives the progressive deterioration of macro-renal function in clinical settings.

The incorporation of real-time metabolic monitoring is increasingly central to organoid-based disease modeling. Barroso et al. [172] reviewed the application of fluorescence lifetime imaging microscopy and phosphorescence lifetime imaging microscopy for organoids, highlighting their ability to non-invasively capture intracellular cofactor states, such as NAD(P)H free/bound ratios, and oxygen distribution, facilitating spatiotemporal resolution of metabolic heterogeneity. Liu et al. [173] extended this approach by combining impedance-based biosensing with real-time imaging in basal stem cell-derived olfactory epithelium organoids to investigate Alzheimer's disease (AD)-associated olfactory dysfunction. Their multimodal platform continuously recorded organoid proliferation, network formation, and structural dynamics, including protein aggregation such as Aβ plaques, enabling real-time tracking of functional and morphological evolution in neurodegenerative disease models. Clinically, the gold standard for diagnosing AD relies on cerebrospinal fluid Aβ-42 and Tau protein assays combined with positron emission tomography imaging. In contrast, this chip utilizes impedance sensing to capture the degradation of peripheral olfactory epithelial neural circuits induced by Aβ deposition, directly mapping onto the underlying pathological essence of peripheral olfactory circuit degeneration observed in AD patients. Because the onset of this functional abnormality occurs far earlier than macroscopic brain atrophy manifests on imaging, this platform provides a highly sensitive timeline to predict AD progression.

Finally, machine learning has been integrated with organoid monitoring to enhance throughput and predictive capacity. Ferreira et al. [174] developed a pancreatic ductal adenocarcinoma (PDAC) organoid-peripheral blood mononuclear cell (PBMC) co-culture system integrated with the deep-learning tool OrganoIDNet, enabling continuous imaging-based quantification of organoid morphology, growth, and heterogeneity in response to therapy. To resolve the critical challenges of cell motility and focal plane instability during longitudinal multicellular imaging, the authors designed an optimized sandwich-based co-culture protocol. This structured hydrogel configuration provided essential spatial confinement to guarantee a stable Z-position for high-resolution image acquisition without hindering PBMC migration, thereby delivering clear data feeds for AI recognition. Functioning as a virtual digital sensor, the OrganoIDNet algorithm automatically segments and identifies PDAC organoids over extended durations, continuously tracking volume changes and precisely measuring eccentricity. Through dynamic pixel and texture analysis, the algorithm non-invasively distinguishes between healthy and unhealthy states, achieving quasi-real-time cell viability tracking analogous to chemical probes without cell destruction. To validate the predictive accuracy of these computer-vision profiles, the resultant digitized continuous curves were cross-benchmarked against traditional endpoint destructive luminescent assays, such as CellTiter-Glo. While the corroborating chemical assay inherently represents a terminal, cell-lytic readout, the deep-learning approach successfully demonstrated the capacity to mirror these destructive endpoints non-invasively throughout the longitudinal culture. Consequently, this convolutional neural network algorithm standardizes high-temporal-resolution phenotypic tracking and transforms previously subjective morphological assessments into high-throughput, reproducible data. Overall, the integration of machine learning with live-cell imaging significantly enhances the analytical throughput and phenotypic tracking capacity of organoid-based disease monitoring. Clinically, the gold standard for assessing pancreatic cancer progression relies on computed tomography (CT) or MRI based on RECIST to evaluate changes in tumor volume and eccentricity. The dynamic volume and eccentricity curves of PDAC organoids extracted by the OrganoIDNet algorithm precisely simulate the underlying principles of evaluating tumor progression via CT or MRI imaging. Furthermore, this algorithmic tracking demonstrates a highly linear correlation with conventional destructive laboratory assays such as CellTiter-Glo, enabling the real-time, non-destructive image-based capture of malignant cancer cell migration and morphological heterogeneity evolution.

### 4.3 Drug screening and personalized therapeutics

**Fig. 8:**
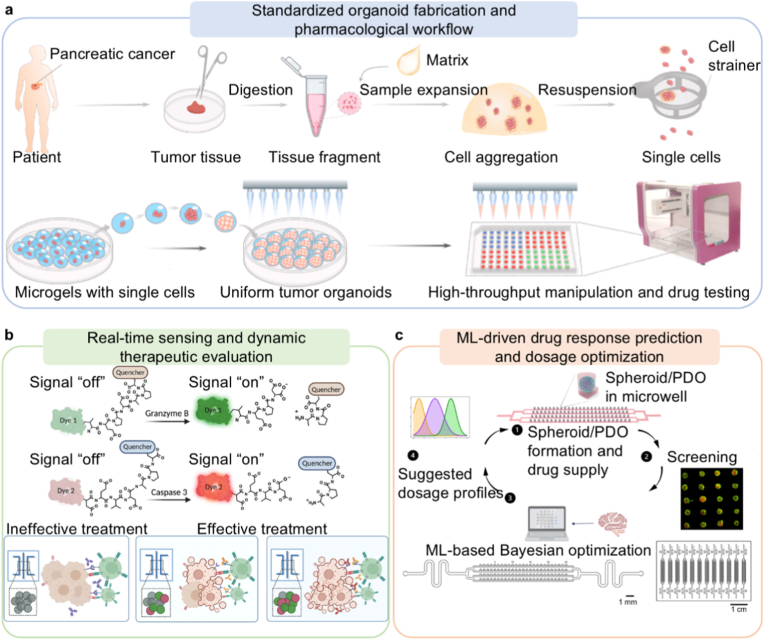
Applications of sensing-integrated organoid-on-a-chip in drug screening and personalized medicine. (a) Standardized organoid processing pipelines, illustrating the full-stage workflow from pancreatic cancer tissue digestion and single-cell microgel droplet generation to uniform tumor organoid maturation and high-throughput drug testing [160], copyright 2023, Elsevier, license: 6285841489122; (b) Real-time sensing trajectories for immunotherapeutic evaluation, showing the molecular “off-to-on” fluorescence switching mechanism mediated by peptide cleavage under Granzyme B and Caspase 3 pathways to differentiate ineffective from effective treatments [179], copyright 2024, John Wiley and Sons, license: 6285850634879; (c) Machine learning-driven drug response prediction networks, demonstrating closed-loop dosage profiles optimized via algorithmic BO coupled with microfluidic multi-well spheroid arrays [180], published under CC BY-NC license.

The integration of physiologically relevant organoid models into drug development pipelines has accelerated therapeutic evaluation across both mechanism-based and clinically-oriented screening. In renal drug evaluation, foundational studies have moved beyond structural mimicry to establish quantitative, mechanism-based indicators of therapeutic responses. Musah et al. [175] dentified glomerular protein deposition and extracellular matrix remodeling, such as C3 and VCAM1 expression, as critical readouts for monitoring kidney injury and chronic disease progression in vitro. Leveraging these mechanistic benchmarks, PDOs generated from fresh, pre-treatment biopsies successfully bridge in vitro drug sensitivity with definitive clinical parameters, such as RECIST or pCR [169]. Expanding these clinical boundaries, Shih et al. [176] employed live imaging to track real-time cytotoxicity and immune-mediated killing in breast cancer organoids treated with chemo- and immunotherapies, while Broutier et al. [177] utilized liver cancer organoid-on-chip systems to validate the translational utility of ERK inhibitors through functional assays like CYP450 expression and ALB secretion. As these biological screening scales shift from isolated laboratory anomalies to massive, multi-lineage datasets, the traditional reliance on such destructive endpoint assays and time-lapse optical imaging creates an unsustainable throughput mismatch. Indeed, advanced droplet-based microfluidics [178] and 384-well automated arrays [160] have standardized the production of uniform pancreatic and liver organoids for high-throughput toxicity assessment (Fig. 8a). The scaling enables rapid ASO screening within a two-month window [181], drives massive “mini-nephron” campaigns screening nearly 10000 organoids [182], and powers multi-organoid platforms [183] to resolve systemic, cross-tissue viral dynamics. Complementing this physical scaling, CRISPR-based functional genomics [184] and scRNA-seq further map high-dimensional gene-to-phenotype trajectories, linking genetic perturbations directly to regulatory network reprogramming for novel target identification. As the personalized medical applications of organoid-on-a-chip technologies expand from high-fidelity disease modeling toward mass-scale screening, the traditional reliance on destructive endpoint assays and time-lapse optical imaging has driven an unsustainable technological mismatch. Bridging this gap requires integrating analytical sensors directly into the microfluidic architecture to continuously stream functional metrics in situ.

Within pipelines for novel drug development and personalized oncology, for instance, Liu et al. [23] reported an approach integrating biosensors into organoid-on-chip platforms, enabling continuous characterization of biomechanical, biophysical, and biochemical parameters. This system provides a reliable platform for longitudinal monitoring of drug mechanisms and real-time assessment of high-throughput pharmacological effects. Yue Yan et al. [185] addressed a critical clinical paradox by developing a patient-specific bladder cancer organoid biosensing platform, where chemotherapy potentially drives treatment resistance and tumor metastasis. To capture this transition non-invasively, they integrated an intracellular oligonucleotide-functionalized nano-biosensor featuring probes modified with a fluorophore and an oligonucleotide strand complementary to delta-like canonical Notch ligand 4 (DLL4) mRNA. While fluorescence is quenched by the substrate in the unbound state, spontaneous cellular internalization and specific hybridization with the target DLL4 mRNA trigger a conformational change that linearly translates the intracellular transcript abundance into a real-time dynamic fluorescence signal. Coupled with 3D time-lapse microscopy, this approach achieves tomographic sensing to precisely identify localized cell populations within the organoid, such as those situated at the invasive frontier, that pioneer DLL4 gene activation. Crucially, the platform engineered a tumor-on-gel biomimetic microenvironment rather than a fully embedded matrix. This structural material design establishes definitive physical boundaries and provides essential 3D support, effectively stabilizing freshly isolated patient-derived tumor organoids to preserve their native architecture prior to chemotherapeutic exposure, while concurrently serving as a physical scaffolding that guides isolated cancer cells and cancer-associated fibroblasts to self-assemble into 3D microtumors. Furthermore, this biomimetic hydrogel substrate functions as an active-matrix runway that accommodates the dynamic infiltration of malignant cells. By utilizing 3D time-lapse confocal microscopy, the platform achieves continuous, in situ tracking of precise invasion depths and development in real time throughout the entire course of cisplatin exposure, delivering a live phenotypic history rather than a static endpoint measurement. Clinically, assessing the risk of recurrence and invasion in bladder cancer post-resection or post-chemotherapy relies on cystoscopy combined with multi-point tissue biopsies as the gold standard. In contrast, this platform captures the dynamics of microscopic cell populations at the invasive front via DLL4 mRNA sensing. The in situ captured surge in DLL4 transcription represents the exact molecular origin driving cancer cells to undergo epithelial-mesenchymal transition and penetrate the basement membrane, thereby mapping onto the histopathological evolution used in clinical settings to evaluate bladder cancer invasion depth (T staging) at the molecular level. Simultaneously, this microscopic molecular monitoring enables the early prediction of chemotherapy-induced malignant progression risks well before visible invasion manifests in macroscopic tissues. In immuno-oncology drug screening, real-time assessment of organoid responses to immune checkpoint inhibitors (ICIs) is essential for early evaluation of therapeutic efficacy and detection of resistance mechanisms. Nguyen et al. [179] engineered a microfluidic organoid-on-chip platform integrated with an activatable dual-sensing nanoreporter system (GCNR), utilizing an amphiphilic polymer backbone monolithically conjugated with two distinct, spectrally orthogonal fluorescent dye-quencher pairs (Fig. 8b). This dual-functional material design uniquely addresses a critical biological ambiguity: despite successful delivery of granzyme B (GrzB), downstream caspase-3 (Casp3) activation can be arrested by intracellular anti-apoptotic proteins, or conversely, Casp3 activation can be initiated via alternative death-receptor pathways independent of GrzB. Consequently, simultaneous tracking of both proteases is indispensable to verify authentic cytotoxic execution. The sensing mechanism relies on engineered, enzyme-specific cleavage sites embedded within the polymer network; upon internalizing into tumor cells within the 3D matrix, active GrzB and Casp3 independently cleave their respective peptide linkers to disrupt the dye-quencher proximity, thereby translating the dynamic kinetics of the intracellular protease cascade into real-time, non-destructive fluorescent signals. Tracking tens of organoids per channel continuously for 48-72 h, this integrated platform enables high-throughput, longitudinal profiling of hot and cold tumor dynamics to accelerate the evaluation of single or combinatory ICI regimens and facilitate the early elucidation of immune resistance mechanisms. Addressing a critical clinical bottleneck, the evaluation of immunotherapy efficacy conventionally relies on measuring GrzB levels released by activated T cells in peripheral blood or the tumor microenvironment, combined with subsequent CT imaging based on RECIST. However, the clinical occurrence of hyperprogression or pseudoprogression frequently leads to misinterpretations in macroscopic imaging. To circumvent this imaging lag, the real-time tracking of intracellular GrzB release and caspase-3 activation via the dual-fluorescent nanoprobe on this platform distinguishes true immune responses from cell-mediated drug resistance well before macroscopic imaging alterations manifest, thereby providing timely decision-making guidance for combinatory immunotherapy regimens. Yakavets et al. [180] advanced high-throughput phenotypic monitoring by establishing an automated, closed-loop platform that integrates large-scale microfluidic arrays with Bayesian optimization (BO) to accelerate multi-drug regimen discovery. To efficiently navigate a complex parameter space encompassing dosage, exposure duration, and administration sequence, the architecture utilizes programmable fluidic routing to apply precise concurrent or time-staggered drug profiles to uniform breast cancer spheroids immobilized within tissue-mimetic hydrogels under interstitial flow. Controlled by the Gryffin algorithm, dynamic phenotypic responses streaming from the microfluidic array serve as real-time feedback to adaptively update the machine-learning model, which batch-proposes optimized conditions for subsequent experimental iterations. This temporal microenvironment-sensing loop successfully decoded scheduling-dependent therapeutic mechanisms, identifying a sequential chemotherapeutic regimen (5-FU, DOX, and CPA) that significantly reduces total dosage while maintaining efficacy, and defining the strict necessity of concurrent supply for specific synergistic drug pairs (Fig. 8c). From a clinical perspective, establishing sequential dosing regimens conventionally requires years of high-risk, high-cost human clinical trials. In contrast, this platform utilizes programmable microfluidic pathways combined with the Gryffin automated closed-loop system to receive real-time phenotypic feedback from organoids, precisely simulating the clinical decision-making logic where physicians dynamically adjust prescriptions based on therapeutic efficacy. By accelerating this clinical trial process in vitro, the system optimizes dosing strategies to achieve identical therapeutic efficacy while substantially reducing the required dosage.

Beyond electrical biosensing, label-free organoid photoacoustic imaging (LFOPI) has been used to non-invasively monitor organoid volume and morphological changes with higher resolution and accuracy than conventional diameter measurements. Luo et al. [186] combined LFOPI with organoid-on-chip platforms to capture structural changes in response to cisplatin, temozolomide, and immunotherapeutics, preserving organoid irregularity and heterogeneity while avoiding potential interference from traditional labeling methods. From the perspective of material design and device integration, the LFOPI system elegantly achieves the heterogeneous integration of microfluidic chip substrates with high-performance acoustic sensing components. By employing materials characterized by low acoustic attenuation and high acoustic transmittance as the coupling media, and incorporating a high-frequency LiNbO_3_-/PZT-based piezoelectric ultrasonic transducer, the system enables highly sensitive acoustic detection and maximizes signal collection efficiency without disrupting the microfluidic environment. This approach provides physiologically relevant, real-time functional readouts for high-accuracy drug screening. Clinically, due to the highly irregular shapes of malignant gliomas, physicians evaluating the efficacy of temozolomide must rely on MRI for 3D tumor volume reconstruction rather than a single diameter measurement. In response, this platform introduces the LFOPI system, utilizing high-frequency piezoelectric ultrasound transducers to capture faint acoustic signals and simulate the imaging principles of clinical radiology for evaluating tumor deformation. Combined with in vitro organoid modeling, this approach recapitulates the highly complex spatial irregularity and morphological heterogeneity of the tumor, demonstrating a high linear correlation with the objective response rate observed in post-chemotherapy clinical pathology.

Automated bioreactor systems also facilitate high-throughput pharmacological assessment. Xie et al. [187] fabricated uniform, biomimetic core-shell skin organoids within spinning bioreactors. The core consists of a Type I collagen hydrogel network encapsulating human dermal fibroblasts to mimic the dermal extracellular matrix, while primary human keratinocytes are seeded onto this core to self-assemble into a stratified epidermal shell. The biomimetic dermal matrix provides the critical biophysical compliance and structural biocompatibility required to guide primary keratinocytes toward stratified terminal differentiation. Structurally, the controlled fluid shear stress generated by the rotating bioreactor drives the uniform formation and terminal differentiation of these bilayered constructs. Mechanistically, the platform couples this engineered microenvironment with a genetically encoded Wnt/β-catenin luciferase reporter system integrated into the epidermal layer. Upon pathway activation by bioactive compounds, accumulated β-catenin translocates into the nucleus to drive luciferase expression, thereby converting abstract biochemical stimuli into quantifiable bioluminescent signals. Under high-calcium spinning cultivation, the scaffolding framework promotes the formation of a mature stratum corneum barrier that tightly regulates chemical permeability, ensuring that the endogenously monitored reporter kinetics reflect authentic tissue-level pharmacological dynamics rather than noise artifacts stemming from dye leakage. Adapted into a 96-well format for high-throughput screening, this sensor-integrated platform enables the simultaneous evaluation of dynamic Wnt signaling kinetics, cell proliferation via Ki67, apoptosis via cleaved-caspase-3, and barrier maturation based on E-cadherin and vimentin expression. The successful identification of minoxidil validates the utility of these sensor-functionalized 3D organoids for automated, high-throughput functional drug screening. Clinically, evaluating the hair growth promotion efficacy of drugs such as minoxidil relies on biopsy histopathological sections, frequently using immunohistochemistry to detect hair follicle matrix activity through Ki67 and barrier protein E-cadherin expression. Guided by this clinical assessment modality, this chip utilizes luciferase to translate Wnt signaling pathway activation into optical dynamic curves. Furthermore, through direct on-chip in situ staining validation, the platform establishes a linear correlation between real-time in vitro optical signals and functional protein expression levels, overcoming the limitation of clinical biopsies that cannot continuously track the dynamic variations in individual drug efficacy.

Toxicity evaluation remains a critical component of drug development. Koichiro Susa et al. [188] reported a renal organoid platform integrating a genetically encoded ATP/ADP biosensor to monitor segment-specific nephrotoxicity. Utilizing stem cell self-assembly, this platform reconstructs continuous epithelial structures containing podocytes, proximal tubules, loops of Henle, and distal nephrons, expressing critical transporters like OAT1, OAT3, and OCT2 to replicate the physiological transport microenvironment missing in 2D cultures. Mechanistically, the platform couples this transport microenvironment with biosensing by embedding a protein-conformational ATP/ADP sensor into the cells to translate intracellular metabolic stress into quantifiable optical readouts. When nephrotoxic drugs like tenofovir or aristolochic acid are actively pumped into proximal tubules via these OAT/OCT pathways, the localized accumulation disrupts mitochondrial function and triggers ATP-to-ADP conversion, inducing a biosensor conformational shift and fluorescence depletion. This transport-mediated toxicity and its coupled sensing mechanism are validated when specific inhibitors like probenecid successfully block the transport channels and reverse the signal depletion. Adapted into a 96-well format, this system enables parallel, high-throughput screening across multiple drugs and doses for early nephrotoxicity prediction. This pre-clinical screening capability is vital, as clinical identification of acute kidney injury relies on detecting elevated serum creatinine or blood urea nitrogen, which is mechanistically driven by drug accumulation in proximal renal tubular epithelial cells leading to mitochondrial dysfunction. To recapitulate the process on-chip, this platform utilizes genetically encoded ATP/ADP sensors to track transporter-mediated early mitochondrial stress and energy transients in situ at the live-cell metabolic level. By translating metabolic impairment into fluorescence fluctuations, this platform directly maps onto the early triggering molecular mechanisms of clinical acute kidney injury.

In summary, the evolutionary transition of organoid platforms from structural mimics to sensor-integrated analytical tools has redefined the boundaries of preclinical drug assessment. By embedding non-invasive biophysical and biochemical transducers directly into multi-segmented tissue microenvironments, these systems bridge the historical gap between endpoint molecular assays and dynamic physiological execution. However, establishing these platforms as validated pharmacological standards requires overcoming several critical engineering barriers. The depth-dependent reliance of photoacoustic imaging and fluorescent probes on focal plane alignment, coupled with the profound scattering and attenuation of light within dense tissues, routinely introduces severe measurement artifacts. Furthermore, the prolonged immersion of chip-integrated sensing electrodes or piezoelectric transducers in complex, protein-rich biological media triggers substantial biofouling, which induces baseline signal drift, sensitivity degradation, and potential long-term cytotoxicity. From a logistical perspective, scaling these architectures into 384-well arrays managed by Bayesian optimization creates an immense computational and data-handling bottleneck; simultaneously executing high-definition 3D time-lapse imaging and multi-modal continuous sensing often causes data latency and signal skewing, rendering true real-time streaming analytics unfeasible. Finally, platforms utilizing genetically encoded reporters, such as luciferase or ATP/ADP biosensors, are intrinsically constrained by the variations of lentiviral transduction and genome editing. During large-scale expansion, the inability to standardize transduction efficiency, expression stability, and sensing fidelity across disparate organoid batches directly compromises the rigorous reproducibility demanded by high-throughput screening pipelines. Resolving these intertwined material, physical, and genetic constraints represents the next essential paradigm shift to successfully transition sensor-functionalized organoid-on-chip arrays from laboratory breakthroughs to predictable clinical translations.

### 4.4 Neural activity and proto-consciousness monitoring

With the rapid advancement of human brain organoid technology, these in vitro 3D models not only recapitulate molecular and cellular features of brain development but also increasingly exhibit complex neuronal network activity. Recently, researchers have begun exploring the identification and monitoring of potential proto-conscious or consciousness-like states within brain organoids, a critical step for understanding how highly organized neural activity and information integration relate to cognitive functions. Brain organoids are studied using a combination of electrophysiological, optogenetic, and imaging techniques to capture both spontaneous and stimulus-evoked neuronal activity. By analyzing network synchrony, oscillatory patterns, and information entropy, these studies provide quantifiable metrics for proto-conscious states. This line of research offers new perspectives on brain development, neurological disease modeling, and drug screening, positioning it at the forefront of brain organoid research.

**Fig. 9:**
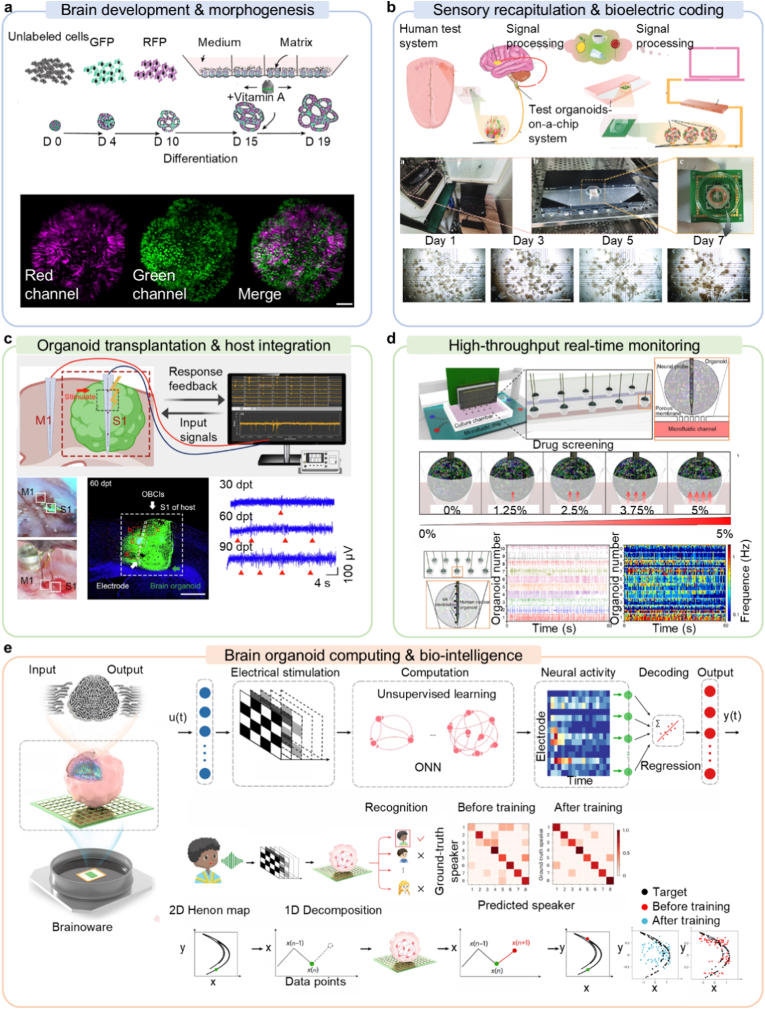
Applications of organoid-on-a-chip for real-time monitoring of neural networks, sensory functions, and biocomputing. (a) Morphological tracking of brain organoid development [189], published under CC BY license, illustrating brain organoid development and spatial neurogenesis profiles from unlabeled progenitors to GFP/RFP co-expressed matrix configurations; (b) Sensory recapitulation and testing, illustrating sensory signal processing and long-term hardware interfaces within testing gustatory organoids-on-a-chip [190], published under CC BY license; (c) Interfacial integration at the organoid-host brain interface, demonstrating in vivo organoid brain-computer interfaces that capture reciprocal input-output response feedback and stable field potential evolution [191], copyright 2024, Springer Nature, license: 6285860086258; (d) High-throughput real-time monitoring and drug screening systems, highlighting automated neural probe microfluidic arrays for parallelized drug screening and concentration-dependent firing frequency heatmap mapping [192], published under CC BY license; (e) Biological reservoir computing systems driven by “Brainoware” paradigms, illustrating unsupervised learning loops via organic neural networks that convert electrical stimulation vectors into decoded outputs, validated through voice recognition (predicted speaker confusion matrices) and nonlinear dynamical systems simulation (2D Hénon map decomposition) [193], copyright 2023, Springer Nature, license: 6285860467244.

Jain et al. [189] recently provided a comprehensive characterization of morphodynamics in early human brain organoid development, laying an essential foundation for future consciousness-related monitoring. Using fluorescently labeled iPSC-derived unguided brain organoids, they performed long-term live light-sheet imaging, combining dual-channel, mosaic, and multi-protein labeling with computational demultiplexing to quantify multiple subcellular structures, such as actin, microtubules, plasma membrane, nucleus, and nuclear envelope, throughout weeks of development. Integration with single-cell RNA sequencing (scRNA-seq) revealed that cellular morphology and organization were tightly coupled to gene expression programs during key stages, including neuroepithelial induction, lumen formation, maturation, and regionalization. Notably, lumen expansion and cell composition were closely linked to ECM regulation and mechanosensing pathways; the use of exogenous matrices like Matrigel enhanced lumen growth and telencephalon formation, whereas organoids grown without matrix tended toward neural crest or caudalized identities. Mechanistically, WNT and Hippo-YAP1 signaling, particularly spatially restricted induction of WLS, played central roles. These findings establish a structural and developmental framework for the subsequent integration of electrical and optical recording technologies to measure neuronal activity and network properties, making the investigation of proto-consciousness and network complexity in organoids increasingly feasible. Sensory organoids provide complementary in vitro platforms for modeling human perception and consciousness-like bioelectrical activity (Fig. 9a). Wu et al. [190] developed a taste organoid-on-chip system by integrating taste organoids with a MEA, faithfully recapitulating key aspects of the human gustatory system. The sensing principle relies on the non-invasive recording of extracellular field potentials triggered by transmembrane ionic fluxes when taste receptors bind to chemical stimuli. Utilizing this platform, the authors confirmed expression of critical taste receptors, such as the acid-sensing channel OTOP1 and the sweet receptor TAS1R2, and recorded extracellular potentials in response to acidic, sweet, bitter, and salty stimuli. Feature extraction and PCA enabled discrimination of taste modalities and stimulus concentrations, demonstrating that sensory organoids encode stimulus-specific bioelectrical patterns. Together with brain organoid studies, these results indicate that organoid platforms can capture bioelectrical correlates of perception, providing a foundation for exploring proto-conscious or consciousness-like processes in vitro (Fig. 9b).

Brain organoids have demonstrated unique potential in neural repair and neural network studies. Nan Hu et al. [191] transplanted brain organoids into damaged brain regions and integrated them with implantable electrodes and stimulation-recording devices. The sensing technology utilizes high-density microelectrode contacts to capture local field potentials and axonal action potentials at the graft-host interface, establishing a functional interface that couples organoid activity with host brain networks. By modulating stimulation parameters, such as frequency and amplitude, the study quantified neuronal firing rates, inter-spike intervals, and synchrony indices, thereby assessing neuronal functionality and network dynamics. Concurrent immunofluorescence analysis of layer-specific markers (SATB2, CTIP2, TBR1), synaptic proteins (SYN, PSD95), and neuronal and astrocytic markers (NeuN, GFAP) allowed evaluation of organoid differentiation, network formation, and astrocyte responses. The results revealed enhanced connectivity and projections between the graft and host brain, alongside promoted organoid maturation and functional integration, highlighting the potential of organoid-brain network interactions and providing a technical foundation for future investigations into proto-conscious or consciousness-related activity (Fig. 9c).

Beyond neural repair, organoid responses to pharmacological or electrical stimulation can offer measurable indicators of consciousness-related activity. Hyogeun Shin et al. [192] developed a “one-step” drug-neural stimulation system. The sensing architecture employs 3D microelectrodes that penetrate the organoid matrix to record extracellular spikes and network synchronization across multiple tissue depths. This configuration establishes a direct biophysical coupling mechanism, where the microfluidic channel continuously regulates the chemical microenvironment, and the 3D-MEA simultaneously captures the resultant metabolic-to-electrical network shifts. The drug-neural stimulation system integrating brain organoids with a microfluidic chip and 3D MEA for real-time monitoring of neuronal firing rate, burst rate, active electrode number, and network synchrony, while simultaneously assessing responses to drugs or external stimuli across multiple organoids. Their findings showed that varying concentrations of potassium chloride significantly enhanced spiking activity and revealed functional differences among organoids. In disease modeling and perception studies, patient-derived SCN2A mutant organoids exhibited marked deviations in firing rate, burst activity, and network synchrony compared with controls, demonstrating genotype-specific functional phenotypes. This platform enables high-fidelity capture of organoid neural dynamics and stimulus responses, establishing a reliable experimental basis for exploring proto-conscious or consciousness-related neural activity (Fig. 9d). However, the inherent compositional volatility and batch-to-batch variations of conventional basement matrices like Matrigel introduce significant confounding noise into high-throughput electrophysiological sensing. This material-induced heterogeneity drives asynchronous cortical development and heterogeneous neural maturation, which manifests as highly erratic baseline firing frequencies across individual brain organoids. In three-dimensional microelectrode array profiling, these baseline variations artificially distort the captured fold-change responses to pharmacological stimulants. Consequently, this material-derived interpretation noise obscures genuine drug efficacy and blurs the boundary between true therapeutic action and matrix-induced artifacts. Circumventing the matrix-induced artifacts requires an improvement toward well-defined engineered scaffolds, such as silk-based biomimetic networks, to stabilize baseline neural activity and secure reproducible electrophysiological readouts.

Building on brain organoid neural functionality and consciousness potential, these systems have been further leveraged for brain-inspired computing. Cai et al. [193] cultured brain organoids on high-density MEAs to form input-organoid-output neural network systems, positioning the organoids as core components of a biological reservoir computing framework. The physical sensing and stimulation mechanism utilizes spatiotemporal electrical pulse trains to recruit neural ensembles, tracking real-time spiking activity and post-stimulation decay to decode network plasticity. By applying spatiotemporal electrical stimulation, the organoids exhibited nonlinear dynamics and echo state properties. The platform enabled real-time recording of spiking activity, response rates, and post-stimulation decay, while also revealing network plasticity and functional connectivity remodeling. Experiments demonstrated that variations in stimulation parameters elicited nonlinearly enhanced organoid firing responses, and after training, the organoid network achieved improved performance in tasks such as speech recognition, with accuracy increasing from ∼51% to 78% (Fig. 9e). However, the structural reliance on conventional flat, rigid MEA substrates introduces a severe spatial and mechanical boundary mismatch against the ultra-soft, 3D organoid matrix. The rigid-to-soft interface restricts electronic communication to a superficial layer of surface neurons, leaving the vast computational bandwidth of deeper interconnected neural networks largely untapped. Furthermore, the unoptimized mass transport within the underlying Matrigel substrate precipitates localized core hypoxia and cell necrosis, prematurely truncating the operational lifespan of the living hardware. To circumvent the interfacial limits, future iterations must transition toward tissue-conformable, three-dimensional flexible electronics while engineering highly oxygen-permeable, non-disruptive matrix materials customized for chronic electrophysiological coupling.

In routine clinical practice, evaluating brain injury repair, epileptic pathology, or cognitive impairment primarily relies on EEG to capture cortical mass-firing oscillations, alongside fMRI utilizing blood-oxygen-level-dependent effects to map macro-regional brain network connectivity. Crucially, the integrated MEA within this platform series operates as an in vitro microscopic extension of these macroscopic clinical diagnostics. By employing microelectrodes tightly coupled to the organoids, the MEA captures field potential variations and interfacial synaptic transmission from single neurons and localized cell populations, directly corresponding to the underlying bioelectric origins of clinical EEG principles and fMRI network functional connectivity. Furthermore, the neural network synaptic plasticity measured via electrical pulse stimulation successfully translates advanced biological reservoir computing capabilities into quantified bioelectric signal outputs. Logically, this alignment echoes the clinical trend of utilizing cognitive and linguistic scales for the endpoint evaluation of high-level nervous system functional recovery. Consequently, such platforms transcend conventional efficacy and toxicity screenings, serving as a life-digital translational interface that provides in vitro research frameworks and technological methodologies for future biophysical robotics, alternative sensory reconstruction, and biomimetic brain-inspired computing. The core biomedical applications and key technical configurations of diverse sensing-integrated organoid-on-a-chip platforms are summarized in Table 6.

**Table 6:** Structured comparison of sensing-integrated organoid-on-a-chip platforms for diverse biomedical applications.

Monitoring goal	Core applications	Organoid models	Culture systems	Integrated sensing route	Ref
Electro-metabolic coupling and metabolic synchronization	Analyzing the correlation between electrophysiology and metabolic function in multi-chambered cardiac tissues	Cardiac organoids	3D scaffolds with tunable stiffness and anisotropic stress	Microscale sensor arrays; Soft 3D liquid-metal printed electrodes	[135,136]
Biochemical metabolism and developmental inflection points	Optimizing transplantation windows and quality control	Hepatic organoids	Monitoring vascularization and metabolic maturation	Embedded NO sensors and urea sensors	[133]
Neural circuitry and pathological assessment	Inter-organoid regional connectivity	Brain organoids	Self-assembled multi-region connectivity models constructed via surface growth	MEA combined with calcium imaging	[149]
Long-term energy metabolism monitoring	Dynamically tracking tumor growth	Human lung cancer organoids	3D organoid culture systems	DNA tetrahedron-based ATP nano-sensors	[152]
Secretome and microenvironment simulation	Tumor microenvironment simulation	Vascularized tumor organoids	Vascularized tumor organoid chips integrated with real-time ELISA modules	Real-time ELISA online monitoring modules	[170]
Physical-biological signal coupling	Pathological mechanism studies of genetic diseases	PKHD1-mutant renal organoids	Simulating distal nephron fluid flow and shear stress	Mechanotransduction pathway monitoring	[171]
Spatiotemporal metabolic heterogeneity	Tracking morphological and functional evolution of neurodegenerative diseases	Olfactory epithelium organoids	Multimodal real-time monitoring platforms	Impedance-based biosensing combined with FLIM/PLIM imaging	[173]
Intelligent large-scale cultivation	High-throughput real-time monitoring and prediction	PDAC organoid-PBMC co-culture	High-throughput co-culture monitoring systems	OrganoIDNet	[174]
Individualized chemotherapy response	Rapidly quantifying cisplatin efficacy; Elucidating individualized chemotherapy responses	Patient-specific bladder cancer organoids	Personalized platforms incorporating nanosensors	Nanosensors combined with 3D time-lapse microscopy	[185]
Immuno-oncology evaluation	Real-time monitoring of T cell-mediated killing and ICI efficacy	Tumor-derived organoids	Microfluidic organoid-on-chip platforms	Dual nanosensors	[179]
High-throughput drug combination optimization	Predicting optimal drug combinations and dosing strategies	Microfluidic organoid arrays	Microfluidic systems integrated with automated liquid handling	Automated phenotypic imaging combined with BO	[180]
Label-free volume/morphology monitoring	High-precision monitoring of organoid volume changes and drug responses	Tumor organoids	Chip platforms integrated with label-free photoacoustic imaging technology	LFOPI	[186]
Functional drug screening	Screening Wnt/β-catenin signaling pathway activators	Microspheric skin organoids	Rotating bioreactors	Wnt/β-catenin luciferase reporter systems	[187]
Segment-specific nephrotoxicity	Predicting early nephrotoxicity and transporter-mediated injury	Renal organoids	Platforms integrating ATP/ADP biosensors	ATP/ADP biosensors	[188]
Morphodynamic characterization	Establishing structural and developmental frameworks for consciousness-related monitoring	Early human brain organoids	Fluorescently labeled iPSC-derived unguided organoids	Long-term live light-sheet imaging combined with computational demultiplexing	[189]
Bioelectric coding simulation	Mimicking human sensory systems and bioelectrical feature extraction	Taste organoids	Taste organoid-on-chip systems	MEA	[190]
Neural circuitry functional repair	Functional replacement of injured brain regions and network integration	Brain organoids	Transplanted organoid-host brain interface systems	Implantable electrodes and stimulation-recording devices	[191]
Consciousness-related activity monitoring	Assessing pharmacological/electrical stimulation responses and genotype-specific phenotypes	Brain organoids	One-step drug-neural stimulation systems integrated with microfluidic chips	3D MEA	[192]
Biological reservoir computing	Constructing brain-inspired computing systems	Brain organoids	High-density MEAs	Spatiotemporal electrical stimulation and real-time neural activity recording	[192,193]

However, the temporal fluctuations of complex neural network signals within brain organoids and the emergent manifestation of primitive consciousness have triggered profound ethical discussions. Within the organoid-on-a-chip platforms discussed in this review, real-time sensing technologies, encompassing high-precision data output and decoding, stringent signal quality mandates, and integrated closed-loop regulation techniques, have actively exacerbated the ethical dilemmas. First, the high-precision decoding of brain organoid data via advanced sensor technology demonstrates that in vitro tissues have already exhibited baseline autonomous consciousness, trapping research within an ethical paradox of informed progression. Second, the rigorous technical requirements for high-quality, uncompromised sensor signals preclude the introduction of confounding factors such as anesthesia or analgesia during active stimulation; consequently, potentially sentient brain organoids are compelled to endure high-frequency electrical stimulation while fully awake and inherently incapable of expressing autonomous consent, directly violating the principle of non-instrumentalization in bioethics. Lastly, the closed-loop regulation of the chip establishes a continuous cycle of “sensing readout—systemic analysis—adaptive stimulation write-in”, which forces the brain organoid to perform high-intensity computational labor, thereby depriving the organoid of its moral freedom to maintain quiescence and silence.

Conversely, the three core technologies of real-time sensing organoid-on-a-chip platforms also provide rigorous scientific means to resolve these ethical dilemmas and align with universally accepted animal experimentation ethics standards. First, the high-precision data output can serve as an objective benchmark for determining humane endpoints; specifically, we can establish explicit experimental endpoints by monitoring electrophysiological signals, such as changes in local field potential frequency or amplitude, thereby preventing indiscriminate, blind stimulation. Second, utilizing multi-modal stimulation-sensing technologies allows researchers to safeguard organoid welfare while maintaining high-quality monitoring; for instance, borrowing from clinical transcutaneous electrical nerve stimulation techniques, high-frequency harmless background currents can be introduced to counteract potential distress signals, or optical monitoring can be substituted when anesthetics are administered. Lastly, the on-chip closed-loop regulation system can be engineered with built-in safety thresholds; upon detecting the gradual attenuation or fatigue of the brain organoid's neural signals, the system can autonomously downregulate stimulation intensity or transition into a resting state. Therefore, in the application of brain organoids, the real-time sensing-integrated organoid-on-a-chip platform serves not only as a technological catalyst that prompts moral scrutiny but also as a foundational underlying tool that delivers quantified regulatory pathways and compliance solutions.

## 5 Conclusions and perspectives

This review provides a systematic synthesis of the recent advancements in real-time sensing-integrated organoid-on-a-chip platforms within the field of biomedical research. By synergistically integrating microfluidic hydrodynamic regulation, functionalized biomaterial design, and multidimensional physical or chemical stimulation, researchers have successfully enhanced the physiological fidelity of organoid models, enabling the precise recapitulation of developmental trajectories, functional maturation, and pathological progression. Crucially, a cross-scale synthesis of these advancements yields three core conceptual insights that define the current state of the art. First, from a micro-engineering perspective, the fluidic and physicochemical environments strictly dictate the physiological fidelity and application ceilings of these models; in particular, the heterotypic micro-interface between the culture matrix and the sensing material governs fundamental charge transfer, optical refraction, and mechanical compliance, thereby establishing the critical boundaries for sensor output quality. Second, the seamless integration of multimodal electrical, optical, and mechanical sensing networks has fundamentally reshaped the bioanalytical modality of in vitro testing, replacing traditional destructive, single-timepoint endpoint assays with completely non-invasive, continuous, and dynamic functional tracking. Third, across diverse downstream applications spanning developmental biology, disease modeling, drug screening, and cutting-edge neural exploration, the in situ output of cross-scale sensing datasets, including real-time chemical flux, high-frequency electrophysiology, and micro-nano mechanical vectors, is increasingly achieving point-to-point alignment with clinical gold standards, including blood biochemical profiles, ECG/EEG waveforms, and macroscopic imaging features. Driven by these collective takeaways, current organoid-on-a-chip platforms significantly accelerate the application of these systems in critical preclinical domains.

Despite the promising applications of sensing-integrated organoid-on-a-chip platforms, several critical device-level technical barriers and micro-engineering challenges must be addressed to facilitate their broad biomedical translation. These immediate limits primarily govern the physical hardware architecture, bio-material interfaces, and real-time sensing pathways. First, regarding the data acquisition mode limitations, current systems are predominantly restricted to passive data acquisition, lacking capabilities for real-time active intervention; therefore, future research should prioritize the development of closed-loop regulation systems equipped with adaptive feedback mechanisms that autonomously adjust fluidic perfusion, oxygenation, or stimulation intensities based on real-time sensor streams to enable automated error correction. Second, to resolve insufficient structural complexity of current organoid model and the subsequent lack of physiological fidelity, efforts must be directed toward engineering high-order, multi-lineage assembloids; achieving system-level in vitro functional coupling through the integrated orchestration of functionalized vascular networks, immune cell populations, and neural innervation is essential to recapitulate human physiological homeostasis. Third, to counteract the interfacial failure encountered of sensors during prolonged cultivation, future innovations must implement interfacial material optimization by optimizing the physical structure of interfacial materials or engineering selective adhesion at the sensing-culture matrix interface, such as the application of zwitterionic coatings or PEGylation, which is paramount to prevent the severe bio-fouling during long-term, months-long continuous culture processes. Crucially, the routine deployment of these systems is heavily bottlenecked by the “small chip, large periphery” dilemma. Due to the bulky external pump sources and tangled multi-channel tubing, the entire peripheral setup inevitably occupies most of the incubator space. Furthermore, multi-channel electrochemical or electrophysiological sensing relies on a dense array of rigid shielded cables routed to external workstations, which not only causes severe physical crowding but also introduces electromagnetic noise and motion artifacts during routine incubator operations. To disrupt this cumbersome hardware architecture, researchers can employ programmable passive rocking platforms to periodically alter the chip's tilt angle, leveraging hydrostatic pressure differentials between reservoirs to drive dynamic perfusion. Alternatively, embedding pneumatic micro-valves or micropump arrays directly within the chip layers, controlled via micro-solenoid valve arrays on the chip manifold, can completely eliminate bulky external syringe pumps. Simultaneously, signal transmission constraints can be effectively resolved by introducing chip-scale, low-power wireless transmission modules and developing flexible diaphragms capable of wirelessly transmitting functional data via minute fluidic pressure waves, thereby safeguarding long-term monitoring stability while minimizing the overall instrument footprint. Sixth, to resolve the massive multimodal data decoding bottlenecks where long-term, uninterrupted multi-channel monitoring generates overwhelming continuous data streams, rendering the manual extraction of transient pathological signatures extremely difficult, the field must accelerate the deep integration of organoid technology with AI.

Specifically, multiple breakthroughs demonstrate that deep learning enables efficient imaging data extraction in intelligent hardware. Deloria et al. [194] paired multimodal OC-PAM with radiomics for automated organoid viability classification and rare resistant cluster identification. Zhao et al. [195] developed PhaseFIT, using generative AI to transform phase-contrast images into virtual fluorescence for high-throughput phenotyping. Concurrently, Kim et al. [196] embedded YOLOv8-nano in alveolar models to extract micro-morphological features and track fibrosis progression. Integrating these data streams profoundly elevates the cross-scale predictive power of organoid models. Mo et al. [197] systematically reviewed these algorithmic applications, highlighting OrBITS/HSLCI pipelines for label-free shape tracking and super-resistant cell warning; Elastic Network and LASSO-SVM for personalized drug matching and survival mapping; and scVelo/Monocle3/Slingshot for predicting stem cell trajectories via mRNA velocity. For clinical translation, Bai et al. [198,199] established “AI-Enabled Organoids”, where the Transformer-based ODFormer system accurately predicted patient PFS and OS. Meanwhile, Ramesan et al. [200] combined machine learning with mechanistic mathematical models for in silico perturbations and causal discovery. AI integration now extends comprehensively from downstream analysis to biofabrication and control loops. For quality control, Lee [201] integrated bioprinting with AI for online cell viability monitoring and geometric feedback. For microfluidic control, Asadi Jozani et al. [202] combined organoids-on-a-chip with active learning to build an automated, “self-driving” nanomedicine closed-loop feedback control system. Additionally, Wang et al. [203]proposed “AI-enabled living organoids” for full-lifecycle monitoring, which achieves a biological-level closed-loop feedback control system in organoid intelligence defined by Smirnova [204] via reservoir computing and biofeedback loops. Synthesizing these multi-modal algorithmic streams effectively elevates traditional monitoring frameworks into autonomous, closed-loop diagnostic engines, thereby enabling real-time feedback and self-driving interventions at the living bio-interface.

Beyond these localized technical and hardware barriers, the successful macro-scale translation of these platforms into routine clinical and industrial workflows hinges on addressing broader ecosystem deployment and practical implementation challenges. First, regarding operational costs and adoption thresholds, the inherent reliance on bulky instrumentation, intricate operational conditions, and exorbitant maintenance costs remains a major barrier to routine deployment in non-specialized laboratories, directly restricting the scalability required to displace animal testing in industrial tech-transfer or massive lead compound screening. Overcoming these economic and logistical hurdles necessitates the process optimization and standardization of automated microfluidic arrays. Consequently, engineering lightweight, miniaturized, and highly integrated system configurations, spearheaded by simplifying the user interface through automated, “plug-and-play”, and lightweight fluidic and sensing modules, will be critically essential to minimize instrument footprints and lower operational thresholds. Concurrently, to circumvent potential physical failures of the hardware, a two-stage pipeline can be implemented where clinical sites are solely responsible for primary tissue acquisition and rapid cold chain transport using preservation solutions. Follow up procedures such as tissue dissociation, chip seeding, and continuous sensing are then entirely executed at a centralized facility. This approach ensures that the micro and nano structured sensor integrated organoid chips remain in a stable environment throughout the entire workflow while successfully achieving a sample to result turnaround of merely 48 to 72 h, thereby delivering actionable data within the golden therapeutic window of oncology. To pragmatically meet this tight window across geodistally separated entities, the off-site transport segment is restricted to the initial 4 to 12 h to minimize ischemic degradation, leaving the remaining 36 to 60 h for centralized cell seeding and immediate sensing to capture acute drug-induced alterations during the early post-seeding phase rather than awaiting full organoid maturation [202], a synchronized workflow successfully validated in multi-center clinical trials like the landmark EXALT study [205]. Second, achieving robust industrial standardization and scalability in mass production is essential to mitigate inter-batch variability in both micro-engineered chips and biological matrices. This requires a strategic transition away from conventional PDMS prototyping, which suffers from severe non-specific absorption of hydrophobic drugs and target molecules, toward medical-grade thermoplastics via high-precision injection molding. Establishing standardized manufacturing protocols and quality control benchmarks for hydrogel composition and sensor performance is necessary to ensure experimental reproducibility across different centers. Finally, clear regulatory frameworks and ethical guidelines must be established. As organoid models move closer to clinical decision-making, such as in individualized therapy prediction, defining validation standards for “fit-for-purpose” models and addressing the ethical implications of complex neural or multi-organ systems will be paramount for their broad biomedical acceptance.

In summary, clearing these interfacial, hardware, and logistical limits requires a steady, step-by-step technical progression. To chart the future trajectory, Table 7 chronologically categorizes the primary engineering barriers and feasible solutions across short-, mid-, and long-term horizons, offering a strategic guide for next-generation sensing-integrated organoid platforms.

**Table 7:** Comprehensive architecture of organoid-on-a-chip scaling from localized technical bottlenecks to phased translation methodologies.

Phased horizons	Target bottleneck	Specific engineering problems	Feasible technical solutions
Short-term phase	Fabrication and matrix variabilities	Micro-engineered chips and biological matrices exhibit profound inter-batch variability; conventional PDMS chips suffer from severe non-specific absorption of hydrophobic drugs and target molecules.	Shift from conventional PDMS prototyping toward high-precision injection-molded medical-grade thermoplastics, and establish quality control benchmarks for chemically defined synthetic hydrogels.
Short-term phase	Hardware and sensor instability	Sensors encounter severe bio-fouling and interfacial failure during long-term, months-long continuous culture processes.	Optimize the physical structure of interfacial materials or engineer selective adhesion at the sensing-culture matrix interface to enhance signal robustness.
Mid-term phase	Insufficient structural complexity	Existing in vitro models typically lack high-order structural complexity, making it challenging to recapitulate the dynamically complex physiological homeostasis of multi-organ systems.	Achieve system-level in vitro functional coupling through the high-order integration of functionalized vascular networks, immune cell populations, and neural innervation.
Mid-term phase	Bulky peripheral bottlenecks	Routine deployment is heavily constrained by the “small chip, large periphery” dilemma, where bulky external syringe pumps and intricate tubing networks occupy the majority of incubator space.	Employ programmable passive rocking platforms utilizing hydrostatic pressure differentials to drive fluidics, or integrate embedded pneumatic micro-valves and micropump arrays in situ within chip layers.
Mid-term phase	Restricted signal transmission	Multi-channel rigid shielded cables lead to extreme physical crowding and introduce severe electromagnetic noise and motion artifacts during routine incubator door operations.	Introduce chip-scale, low-power wireless transmission modules and develop flexible diaphragms capable of wirelessly transmitting functional data via minute fluidic pressure waves.
Mid-term phase	Operational costs and adoption thresholds	The inherent complexity and high operational costs of current platforms pose severe barriers to technology transfer into non-specialized laboratories, limiting the potential for large-scale animal testing replacement.	Standardize manufacturing processes for high-throughput automated microfluidic arrays, simplify user interfaces, and develop lightweight, “plug-and-play” modular configurations.
Long-term phase	Data acquisition mode limitations	Current systems are predominantly restricted to passive data acquisition, lacking capabilities for real-time active intervention and dynamic regulation.	Construct automated networks equipped with adaptive feedback mechanisms to autonomously adjust fluidic perfusion, oxygenation, or electrical stimulation based on real-time sensor streams.
Long-term phase	Massive multimodal data decoding	Long-term, uninterrupted multi-channel monitoring generates massive multimodal datasets, rendering the extraction of transient pathological signatures via traditional manual methods extremely difficult.	Deeply integrate advanced artificial intelligence platforms and deep learning algorithms to achieve automated, high-precision decoding of large-scale, cryptic, and complex pathological signatures.

## Ethics approval and consent to participate

All animal procedures were conducted in accordance with the Guidelines for the Care and Use of Laboratory Animals of Sichuan University and were approved by the Animal Care and Use Committee of West China Hospital, Sichuan University (Approval No. 20220714003).

## CRediT authorship contribution statement

**Chenwei Sun:** Conceptualization, Investigation, Visualization, Writing – original draft. **Guohua Wu:** Data curation, Methodology. **Di Wu:** Formal analysis, Resources. **Qijun Du:** Data curation, Validation. **Qingrui Lu:** Investigation, Validation. **Wenqi Hu:** Investigation, Visualization. **Jiashu Wang:** Investigation, Resources. **Ao Xie:** Data curation, Validation. **Zipeng Yao:** Data curation, Visualization. **Mengjiao Xia:** Formal analysis, Software. **Haijie Hu:** Project administration, Supervision, Writing – review & editing. **Shuqi Wang:** Funding acquisition, Project administration, Supervision, Writing – review & editing.

## Declaration of competing interest

The authors declare that they have no known competing financial interests or personal relationships that could have appeared to influence the work reported in this paper.
